# Expression of Enterocin A in *Saccharomyces cerevisiae*

**DOI:** 10.1007/s12602-025-10686-0

**Published:** 2025-08-30

**Authors:** Michelle Rossouw, Gerhardt Coetzee, Rosemary A. Cripwell, Willem H. van Zyl, Leon M. T. Dicks, Carla L. Ritter, Marinda Viljoen-Bloom

**Affiliations:** 1 Department of Microbiology, Stellenbosch University, Private Bag X1, Matieland, 7602 South Africa; 2 Department of Chemical Engineering, Stellenbosch University, Private Bag X1, Matieland, Stellenbosch, 7602 South Africa

**Keywords:** Class IIa bacteriocins, Enterocin A, Mundticin ST4SA, Plantaricin 423, *Saccharomyces cerevisiae*

## Abstract

Recombinant expression in the yeast *Saccharomyces cerevisiae* offers an alternative approach to developing large-scale production systems for class II bacteriocins from lactic acid bacteria, such as enterocin A, mundticin ST4SA and plantaricin 423. An important consideration for bacteriocin activity is disulphide bond formation: mature mundticin ST4SA has one, and plantaricin 423 and enterocin A each have two disulphide bonds. The native bacteriocin operon typically includes accessory proteins that facilitate disulphide bond formation, but this gene is absent in the enterocin A operon. In this study, the recombinant expression of a codon-optimised gene for enterocin A in *S. cerevisiae*, was compared to that for a codon-optimised plantaricin 423 and mundticin ST4SA, previously successfully expressed in *S. cerevisiae*. Shake flasks delivered more than twofold higher peptide EntA_Opt levels than PlaX_Opt and MunX_Opt, with even higher EntA_Opt expression levels in batch fermentations. However, the bacteriocin activity of plantaricin 423 was considerably lower than that of enterocin A and mundticin ST4SA. It is postulated that this could be a result of incorrect disulphide bond conformation due to the absence of the plantaricin accessory protein, PlaC, in *S. cerevisiae*. Nano-LC-MS/MS analysis showed various post-translational modifications for the peptides, with a greater proportion of EntA_Opt peptides with the correct disulphide bond conformation than for PlaX_Opt. This study demonstrated that *S. cerevisiae* is a promising host to produce recombinant class IIa bacteriocins, particularly enterocin A. However, the co-expression of accessory proteins should be investigated to improve the activity of recombinant plantaricin 423.

**Supplementary Information:**

The online version contains supplementary material available at 10.1007/s12602-025-10686-0.

## Introduction

Bacteriocins represent one of the most important groups of antimicrobials with potential applications in the food industry and human health [1]. Bacteriocins are antimicrobial peptides ribosomally synthesised by bacteria and are classified based on their size, structure, modifications and heat tolerance [2]. Class II bacteriocins are the most abundant antimicrobials produced mainly by lactic acid bacteria, with the class IIa bacteriocins the largest and most comprehensively studied subgroup [3]. The class IIa bacteriocins are active against food spoilage and pathogenic bacteria such as *Listeria monocytogenes*, *Staphylococcus aureus*, *Clostridium perfringens*, *Bacillus cereus* and *Enterococcus faecium* [4]. Several class IIa bacteriocins have been granted GRAS (Generally Regarded As Safe) status and are widely used as food preservatives in dairy and meat products. Examples include pediocin PA-1, prepared as a crude fermentate of *Pediococcus acidilactici* (MicroGARD® and ALTA 2431®) and commercially used to inhibit *L. monocytogenes* [5, 6]. Bactoferm™ F-LC contains freeze-dried cultures of *P*. *acidilactici*, *Lactilactobacillus curvatus* and *Staphylococcus xylosus* that produce sakacin A and pediocin PA-1, and are used as starter cultures in salami to inhibit *L. monocytogenes* [5]. Partially purified sakacin A immobilised on polyethylene-coated paper sheets and enterocin incorporated into edible agar films serve as antimicrobial packaging for meat and cheese products [7, 8]. Given the significant demand for class IIa bacteriocins, strategies to improve peptide production are of commercial importance.

Production of class IIa bacteriocins from their natural producer strain is highly regulated on a molecular level and involves several genes in the bacteriocin operon or gene cluster. The operon usually encodes the pre-peptide, an immunity protein, an ATP-binding cassette (ABC) transporter, accessory proteins for the extracellular translocation of the peptide, accessory disulphide bond modification proteins as well as regulatory proteins [9, 10]. Genes encoding accessory proteins involved in the extracellular transport of bacteriocins, and the formation of disulphide bonds are crucial for producing active peptides. High-level production of class IIa bacteriocins in the native hosts is limited due to the tight regulation of gene expression. In addition, large-scale peptide production by native hosts is expensive, as it requires complex media that complicate downstream peptide purification. The media properties, including amino acid composition, carbon-to-nitrogen ratio, salt concentration, pH, and lactose levels, influence cell biomass and bacteriocin production levels [11].

Heterologous expression in a different host could allow for a simplified and more consistent production of bacteriocins. The yeast *Saccharomyces cerevisiae* is a promising host for producing bacteriocins, as it has GRAS status and has been extensively used to produce and secrete industrial proteins [12]. However, the most significant advantage is that a recombinant bacteriocin will not target the yeast cells. In a previous study, codon-optimised and native variants of *plaA* and *munST4SA* (encoding plantaricin 423 and mundticin ST4SA, respectively) were successfully cloned and expressed in the *S. cerevisiae* Y294 laboratory strain [10]. In both cases, high bacteriocin levels were produced from the structural bacteriocin genes under control of the *S. cerevisiae* MFα signal peptide. Although similar yields were obtained for the two bacteriocins (18.4 mg/L plantaricin 423 and 20.9 mg/L mundticin ST4SA), their specific activity levels differed significantly. The minimum inhibitory concentration (MIC) for mundticin ST4SA (108.52 nM) against *L. monocytogenes* was more than tenfold lower than for plantaricin 423 (1 183.67 nM).

Plantaricin 423 contains four cysteine residues with three different potential disulphide bond conformations, while mundticin ST4SA has only two cysteine residues in one conformation. This raised the question whether the lower plantaricin 423 activity is related to incorrect folding, as disulphide bond accessory proteins are usually present in the operons of class IIa bacteriocins containing two disulphide bonds, and rarely in those containing one disulphide bond [13]. In the bacteriocin operon of the native host (*Lactiplantibacillus plantarum* 423), a disulphide bond accessory protein (PlaC) facilitates correct disulphide bond formation of plantaricin 423 [14]. Enterocin A (produced by *Enterococcus faecium*) and divercin V41 (produced by *Carnobacterium divergens*) are two class IIa bacteriocins that lack a gene for a disulphide bond accessory protein in their bacteriocin operon. Enterocin A comprises 47 amino acids, has two disulphide bonds and is extremely potent against *L. monocytogenes*. However, the low levels of enterocin A secreted by the native host prompted investigations into the heterologous production of the peptide in alternative hosts, including bacterial and yeast species [15–17].

Rossouw et al. [10] demonstrated that *S. cerevisiae* is a promising host for producing plantaricin 423 and mundticin ST4SA, resulting in almost double the peptide yields as the *E. coli* expression system used by Vermeulen et al. [18]. In this study, the production of recombinant enterocin A in *S. cerevisiae* was investigated and compared to that of plantaricin 423 and mundticin ST4SA, also considering disulphide bond requirements. The codon-optimised *entA* gene was cloned into a high-copy episomal expression vector and transformed into the *S. cerevisiae* Y294 strain. The expression and activity of the recombinant enterocin A peptide were evaluated and compared to those of plantaricin 423 and mundticin ST4SA. All three recombinant peptides were characterised using nano-LC-MS/MS and their post-translational modifications (including disulphide bonds) examined. The enterocin-producing strain was evaluated in 1-L bioreactors under batch and fed-batch conditions to increase recombinant peptide yields.

## Materials and Methods

### Strains and Plasmids

The yeast and bacterial strains used in this study are listed in Table 1, and the plasmids in Table 2.

**Table 1:** Microbial strains used and constructed in this study

**Strains**	**Genotype**	**Reference/source**
**Bacterial strains**		
*E. coli* DH5α	*sup*E44 ∆*lac*U169 (Φ80*lacZ*∆M15) *hsd*R17 *rec*A1 *end*A1 *gyr*A96 *thi*−1 *rel*A1	[19]
*L. monocytogenes* EDG-e	Cam^R^	[18]
***S. cerevisiae* strains**		
Y294	MATα *lue*2-3112 *ura*3-52 *his*3 *trp*1-289	ATCC 201160
Y294[MR]	*URA*3 *ENO1*_P_-*MFα1*-*ENO1*_T_	[10]
Y294[PlaX_Opt]	*URA*3 *ENO1*_P_-*MFα1*-*plaA_Opt*-*ENO1*_T_	[10]
Y294[MunX_Opt]	*URA*3 *ENO1*_P_-*MFα1*-*munST4SA_Opt*-*ENO1*_T_	[10]
Y294[EntA_Opt]	*URA*3 *ENO1*_P_- *MFα1*-*entA_Opt*-*ENO1*_T_	This study

*ATCC*, American Type Culture Collection

**Table 2:** Plasmids used and constructed in this study

**Plasmids**	**Genotype**	**Reference/source**
pMR	*bla URA*3 *ENO1*_P_-*MFα1*-*ENO1*_T_	[10]
yBBH1-PlaX_Opt	*bla URA*3 *ENO1*_P_-*MFα1*-*plaA*_Opt-*ENO1*_T_	[10]
yBBH1-MunX_Opt	*bla URA*3 *ENO1*_P_-*MFα1*-*munST4SA*_Opt-*ENO1*_T_	[10]
pMR-EntA_Opt	*bla URA*3 *ENO1*_P_- *MFα1*-*EntA_Opt*-*ENO1*_T_	This study
pUC57-EntA_Opt	*bla entA*_Opt	GenScript®

### Media and Cultivation Conditions

Unless stated otherwise, all media components and reagents were purchased from Merck (Darmstadt, Germany). Plasmid propagation was done in *E. coli* DH5α (Takara Bio Inc., Japan); the transformants were maintained and selected on Luria Bertani (LB) agar containing 100 µg/mL ampicillin. Transformants were routinely cultured at 37 °C in terrific broth (12 g/L tryptone, 24 g/L yeast extract, 4 m/L glycerol, 0.1 M potassium phosphate buffer; pH 7.0) containing 100 µg/mL ampicillin [19]. *Listeria monocytogenes* EDG-e, the indicator strain for antimicrobial activity assays was maintained at 37 °C on brain heart infusion (BHI) agar supplemented with 7.5 µg/mL chloramphenicol.

The *S. cerevisiae* Y294 laboratory strain served as a host for recombinant gene expression, was maintained on YPD agar (10 g/L yeast extract, 20 g/L peptone, 20 g/L glucose, 20 g/L agar), and routinely cultured in YPD broth at 30 °C. Recombinant *S. cerevisiae* Y294 strains were selected and maintained on SC^-URA^ agar [6.7 g/L yeast nitrogen base without amino acids (BD Diagnostic Systems, Maryland, USA), 20 g/L glucose, 1.5 g/L synthetic drop-out medium supplements without uracil (Sigma-Aldrich, Steinheim, Germany) and 20 g/L agar; pH 6.0]. The *S. cerevisiae* Y294 recombinant strains were aerobically cultivated on a rotary shaker (200 rpm) at 30 °C in 125-mL Erlenmeyer flasks containing 20 mL 2× SC^−URA^ broth [20]. Unless stated otherwise, all growth media were inoculated at a final cell count of 1 × 10^6^ CFU/mL.

### Construction of Recombinant Yeast Strains

#### DNA Manipulations

DNA manipulations and *E. coli* transformations were performed using standard protocols [19]. The DNA sequence of the *entA* gene (GenBank accession number AF099088.1) encoding the *E. faecium* mature enterocin A (excluding the leader peptide) was codon-optimised by GenScript® (Piscataway, New Jersey, USA) using the OptimumGene™ algorithm for expression in *S. cerevisiae* (Fig. S1). The *entA*_Opt nucleotide sequence was amplified from plasmid pUC57-EntA_Opt with primers designed for yeast-mediated ligation (YML) (Table S1), using the Gene Amp® PCR System 2400 Thermal Cycler (Perkin Elmer, Waltham, MA, USA) and TaKaRa Ex Taq™ polymerase (Takara Bio Inc.). The PCR product was separated on a 2% (w/v) agarose gel and extracted from the gel by the Zymoclean™ Gel DNA Recovery kit (Zymo Research, Irvine, California, USA). Plasmid pMR, the expression vector for *entA*_Opt containing the *ENO1* cassette with the MFα1 secretion signal (Fig. S2), was linearised with *Xho*I (Inqaba Biotechnological Industries, Pretoria, South Africa) and recovered from a 0.8% (w/v) agarose gel with the Zymoclean™ Gel DNA Recovery kit (Zymo Research).

#### Yeast Transformations

The *entA*_Opt PCR product was co-transformed with the linearised plasmid into electrocompetent *S. cerevisiae* Y294 cells [21] using the Bio-Rad system (GenePluserXcell TM, Bio-Rad, Hercules, California, USA) to construct in-frame fusions via YML. After electroporation, 1 mL of YPDS (YPD supplemented with 1 M sorbitol) was immediately added to the cuvette, incubated at 30 °C for 1 h and plated onto SC^-URA^ plates. Positive transformants were identified by colony-PCR using gene-specific primers (Table S1) and Sanger sequencing (ABI PRISM™ 3100 Genetic Analyser, Central Analytical Facility, Stellenbosch University) of the plasmid DNA isolated from the yeast strain using the High Pure Plasmid Isolation kit (Roche, Mannheim, Germany). The *S. cerevisiae* Y294[PlaX_Opt] and Y294[MunX_Opt] strains [10] that produce the *L. plantarum* plantaricin 423 and *E. mundtii* mundticin ST4SA, respectively, were included as controls. The resulting peptides are named PlaX_Opt and MunX_Opt, respectively, while the peptide produced by Y294[EntA_Opt] was named EntA_Opt. Plasmid maps relevant to the study are illustrated in Figure S2.

### Screening for Antimicrobial Activity

The recombinant yeast strains/transformants were screened for antimicrobial activity against *L. monocytogenes* EDG-e using agar-overlay assays [10]. The strains were grown overnight at 30 °C in test tubes containing 10 mL SC^-URA^ broth; 2 µL of each culture were spotted onto SC^-URA^ agar plates and incubated for 72 h at 30 °C. The plates were overlaid with BHI 0.7% (w/v) agar inoculated with a 1% (v/v) overnight culture of *L. monocytogenes* EDG-e. After incubation at 30 °C for 18 h, the plates were examined for inhibition zones, relative to *S. cerevisiae* Y294 transformed with pMR (no AMP insert) as the negative control. Assays were performed in triplicate, and the average and standard deviation of the diameter of the inhibition zones were recorded.

### Bacteriocin Activity Assays

The bacteriocin activity of the recombinant *S. cerevisiae* Y294 strains was evaluated over 96 h using the agar well-diffusion assay [10]. Erlenmeyer flasks (125 mL) containing 20 mL 2× SC^-URA^ broth were inoculated with 1 × 10^6^ cells/mL of the respective recombinant *S. cerevisiae* strains and grown aerobically on a rotary shaker at 200 rpm for 96 h at 30 °C. The cell-free supernatant (CFS) of each transformant was harvested (1 500 *× g*, 5 min) and filtered through 0.2-µm low-protein binding non-pyrogenic membranes (syringe filters, Pall Life Sciences, NY, USA). To determine antimicrobial activity, twofold serial dilutions of the CFSs were made in sterile phosphate-buffered saline (PBS, pH 7.4), of which 100 µL was loaded into 6-mm wells made into the surface of BHI 1% (w/v) agar inoculated with a 1% (v/v) overnight culture of *L. monocytogenes* EDG-e. The plates were incubated at 30 °C for 18 h and examined for inhibition zones. The antimicrobial activity of the recombinant strain was expressed as arbitrary units per millilitre (AU/mL), corresponding to the reciprocal of the highest dilution that inhibited the growth of indicator strain. To determine the dry cell weight (DCW), the biomass in 1 mL of 96-h cultures was harvested by centrifugation (1 500 *× g* for 5 min), dried overnight at 60 °C and weighed. Bacteriocin activity assays were performed with three biological repeats, and the average and standard deviations were determined.

### Peptide Analysis Using Tricine SDS-PAGE

The CFS of the recombinant yeast strains were lyophilised and dissolved in sterile Milli-Q water to achieve a 20-fold concentration and analysed with tricine SDS-PAGE [10, 22]. Tricine SDS-PAGE analyses were performed in duplicate using the ultra-low range molecular weight marker (Sigma-Aldrich). Both gels were fixed for 20 min in a 25% (v/v) isopropanol and 10% (v/v) acetic acid fixing solution and rinsed three times for 15 min with sterile Milli-Q water. One gel was subjected to Coomassie blue staining [22], followed by silver staining [23]. The other gel was cast in a BHI 0.8% (w/v) agar bilayer (supplemented with 7.5 µg/mL chloramphenicol) inoculated with 1% of overnight culture of *L. monocytogenes* EDG-e, and incubated overnight at 30 °C to assess antimicrobial activity [24].

### Peptide Production in Shake Flasks

#### Peptide Production and Purification

The recombinant *S. cerevisiae* Y294[EntA_Opt], Y294[PlaX_Opt] and Y294[MunX_Opt] strains were cultured in 2× SC^-URA^ broth in 125-mL shake flasks as described above, and the CFS harvested and filter-sterilised. The peptides were precipitated from the respective supernatants with TCA-acetone [10] and dissolved in sterile Milli-Q water. The peptides were purified from the respective supernatant extracts with reverse-phase High-Performance Liquid Chromatography (HPLC) with the Agilent 1260 Infinity HPLC system and the ZORBAX 300SB-C8 column (4.6 × 150 mm, 5-μm particle size) (Agilent, Santa Clara, CA, USA). Sample separation was achieved using linear gradient elution from 10 to 60% solvent B over 25 min (solvent B: acetonitrile + 0.1% (v/v) trifluoroacetic acid [TFA] against solvent A: analytically pure water + 0.1% (v/v) TFA]. Elution profiles were monitored at 230 and 214 nm and collected in 250 µL fractions. Each fraction was tested for activity against *L. monocytogenes* EDG-e using the agar well-diffusion assay.

#### Yield Estimation

The HPLC-purified fractions of EntA_Opt that showed anti-listerial activity were combined, lyophilised and analytically weighed using an XP26 Excellence Plus Micro Balance (Mettler Toledo, Columbus, Ohio, USA). The yield of EntA_Opt was determined as the mean of three biological repeats in triplicate technical repeats and compared to the yields previously obtained for MunX_Opt and PlaX_Opt [10]. The purity of the HPLC-purified EntA_Opt was confirmed with tricine SDS-PAGE analysis and agar overlays. A concentration range (10 µg – 312.5 ng) of bovine serum albumin (BSA) was included as a control for densitometry.

### Minimum Inhibitory Concentrations (MICs)

The MIC of EntA_Opt was determined by following the standard broth microdilution method [25] and compared to those previously reported for PlaX_Opt and MunX_Opt [10]. The percentage inhibition was determined as the amount of bacteriocin that inhibited growth by at least 90%. The MIC values were reported as the means of three biological repeats in triplicate technical repeats.

### Liquid Chromatography and Tandem Mass Spectrometry (LC-MS/MS) Analysis

For improved resolution, the HPLC-purified EntA_Opt, PlaX_Opt and MunX_Opt peptides from the C8 column were loaded on a C18 column under the same conditions described above. For each peptide, 50 µg of protein was injected into the column; the eluate was collected in 290-µL fractions and spotted against *L. monocytogenes* using the agar well-diffusion assay. The combined active fractions underwent LC-MS/MS analysis [10] to confirm the accurate peptide mass and putative disulphide bond locations and conformations for each peptide. A Thermo Scientific Ultimate 3000 RSLC equipped with a C18 trap column (PepMap™; 100Å pore size, 3-µm particle size, 0 75 µm × 20 mm) and a C18 analytical column (Waters nanoEase M/Z Peptide CSH C18 Column; 130-Å pore size, 1.7-µm particle size, 150 µm × 150 mm) were used for the LC analysis. The solvent system entailed loading solvent: 2% acetonitrile in water containing 0.1% formic acid; solvent A: water containing 0.1% formic acid and solvent B: acetonitrile containing 0.1% formic acid. The samples were loaded onto the trap column using the loading solvent at a flow rate of 2 μL/min from a temperature-controlled autosampler set at 7 °C. Loading was performed for 5 min before the sample was eluted onto the analytical column. The flow rate was set to 0.3 µL/min, and the gradient was generated with 5 to 85% solvent B from 5 to 45 min, and 85 to 5% solvent B from 45 to 75 min using chromeleon non-linear gradient 5. Chromatography was performed at 50 °C, and the outflow was delivered to the mass spectrometer through a stainless-steel nano-bore emitter.

Mass spectrometry was performed using an Orbitrap Fusion mass spectrometer (Thermo Scientific, Waltham, MA, USA) with a nanospray flex ionisation source. Data were collected in positive mode with spray voltage set to 1.9 kV and ion transfer capillary set to 275 °C. Spectra were internally calibrated using polysiloxane ions at *m/z* = 445.12. The MS1 scans were performed using the orbitrap detector set at 120 000 resolutions over the scan range of *m/z* 375–1500 with an automatic gain control (AGC) target at 40,000 and a maximum injection time of 50 ms. Data were acquired in profile mode. The MS2 acquisitions were performed using monoisotopic precursor selection for ions with charges +2 to +9 with an error tolerance of approximately 10 ppm. Precursor ions were excluded from fragmentation for 60 s. Precursor ions were selected for fragmentation in higher-energy collisional dissociation (HCD) mode using the quadrupole mass analyser with HCD energy set to 30%. Fragment ions were detected with the orbitrap mass analyser set to 30,000 resolutions. The AGC target was set to 50,000, and the maximum injection time to 100 ms. The data were acquired in centroid mode. The LC-MS/MS data were processed and analysed using the MZmine 2 software [26] and pLink® 2 [27].

The HPLC-purified MunX_Opt sample was analysed with gas chromatography and mass spectrometry (GC-MS) at the Central Analytical Facilities (CAF), Stellenbosch University, to determine if fatty acids were bound to the peptide.

### Peptide Production in Bioreactors

#### Batch Fermentations

The best-performing yeast strain, Y294[EntA_Opt], was selected for bioreactor studies following the methodology outlined in Anane et al. [28]. Starter cultures were prepared from a fresh Y294[EntA_Opt] culture in 10-mL SC^-URA^ test tubes, incubated at 30 °C for 48 h on an orbital shaker. Duplicate 250-mL Erlenmeyer flasks containing 50 mL 2× SC^−URA^ (with 20 g/L glucose) each were inoculated with 500 µL of preculture and incubated at 30 °C with shaking (200 rpm) for 24 h or until A_600_ > 3. These cultures served as inoculum (10% v/v) for bioreactors containing 630 mL 2× SC^−URA^ (20 g/L glucose), resulting in a 700-mL working volume. Foam formation was suppressed with 500 µL/L Antifoam 204 included in the growth medium. The fermentations were conducted in a 1-L NBS Bioflo 110 (New Brunswick Scientific, Edison, NJ, USA). Dissolved oxygen (DO) was measured with a polarographic probe (Mettler Toledo, Columbus, OH, USA). The oxygen level was maintained above 30% saturation using a control loop that linked the agitation rate to dissolved oxygen (DO). A constant aeration rate of 0.8 volume of air per volume of medium per minute (vvm) was maintained. The temperature was maintained at 30 °C, the pH was adjusted to pH 5.5 with 2 N KOH and monitored using a combination glass pH electrode (Mettler Toledo), and the agitation rate varied between 100 and 1000 rpm.

After inoculating the bioreactor with the starter culture, aseptically collected 7-mL samples were taken every 2 h for 24 h. The samples were centrifuged (16 000 × *g* for 2 min), and the supernatant was harvested. One mL supernatant was filter-sterilised with a 0.2-µm low protein-binding syringe filter, and 200 µL was used for bacteriocin assays against *L. monocytogenes* (as described above) to monitor peptide production. The remaining supernatant was filter-sterilised (using a 0.22-µm nylon syringe filter) and analysed with HPLC to quantify glucose, glycerol, acetic acid and ethanol concentrations. The cell pellets were dried overnight at 60 °C and weighed to determine the DCW (g/L). The maximum specific growth rate *µ*_max_ (h^−1^) was defined as the slope of the natural logarithm of the DCW as a function of time of the exponential phase. The biomass yield (*Y*_*X/S*_) at the end of the exponential phase was calculated by subtracting the initial biomass (*X*_0_) from the final biomass (*X*_1_), divided by the amount of substrate (*S*; glucose) consumed during the same time (Eq. 1).

1 $${Y}_{x/s}=\frac{{X}_{1}-{X}_{0}}{{S}_{f}-S}$$

#### Fed-Batch Fermentation

The fed-batch cultivations employed a step-wise exponential glucose feeding regime and were initiated upon glucose exhaustion at the end of the initial batch phase, as determined by HPLC analysis. The setup was similar to the batch cultivations, with a starting volume of 400 mL and a specific growth rate (${\mathit\mu}$) of 50% of *µ*_max_. The fed-batch fermentations were conducted in triplicate for 24 h, and peptide production, DCW, glucose, ethanol, glycerol and acetic acid concentrations were determined as described earlier. The flow rate (*F*) for glucose feeding (100 g/L) was determined with Eq. 2, with *X* representing the biomass at the end of the batch phase, *V* the volume of the batch, *m*_*s*_ the maintenance coefficient, *µ* the specific growth rate, *S*_0_ the substrate concentration in the feed and *S* the residual substrate concentration in the bioreactor [28].

2 $$F\left(t\right)=\frac{XV}{(S_0-S)}.\left(\frac{\mathit{\mu}}{Y_{x/s}}+m_s\right)$$

#### Peptide Purification and Yield Estimation

The Y294[EntA_Opt] supernatant was harvested when maximal peptide production occurred after batch and fed-batch cultivation in the bioreactors. The supernatant was freeze-dried, and the proteins precipitated with TCA-acetone as described above. The pellets were dissolved in sterile MilliQ water and purified with Sep-Pak C18 cartridges with acetonitrile as eluent per the manufacturer’s instructions (Waters Corporations, Milford, MA, USA). The peptide samples were loaded onto the column and eluted with a stepwise increase (10 to 80%) in acetonitrile. At each acetonitrile concentration, the fractions were collected and spotted into wells cut into an agar plate containing *L. monocytogenes* to determine antimicrobial activity and subjected to tricine-SDS-PAGE analysis to determine peptide purity. The fractions containing EntA_Opt were combined, freeze-dried and analytically weighed to determine the peptide yields. The purity of the combined active fractions for both the batch and fed-batch fermentations was confirmed with tricine-SDS-PAGE analysis.

### Nucleotide Sequence Accession Numbers

The codon-optimised gene sequence of enterocin A was submitted to GenBank (accession number OR509503.1).

## Results

Improving the heterologous production of bacteriocins in *S. cerevisiae* can significantly benefit food and health industries. This study investigated the expression of enterocin A in *S. cerevisiae* relative to plantaricin 423 and mundticin ST4SA, considering disulphide bond conformations. Plantaricin 423 and enterocin A contain two disulphide bonds, which can result in various disulphide bond conformations. Genes in the bacteriocin operon of lactic acid bacteria encode disulphide bond accessory proteins that facilitate the correct formation of disulphide bonds. However, this gene is absent in the enterocin A operon (Fig. S3), suggesting that post-translational modification of enterocin A could be more simplified than for plantaricin 423, which could also benefit recombinant expression of enterocin A in *S. cerevisiae*. Tandem mass spectrometry analysis was used to elucidate the different disulphide conformations of the recombinant peptide species (different molecular forms or isoforms) and correlated to their antimicrobial activity against a *L. monocytogenes* indicator strain. The yeast strain with the highest activity was selected for up-scaled bacteriocin production in bioreactors.

### Strain Construction

The *entA* gene of *E. faecium* was codon-optimised for expression in *S. cerevisiae* Y294 (Table 1). The codon optimisation increased the codon bias index (CBI) from 0.07 for *entA* to 0.48 for *entA_Opt*, and the codon adaptation index (CAI) from 0.78 to 0.92 (Fig. S4). The *entA_Opt* gene was cloned into the *ENO1*_P_-*MFα1*-*ENO1*_T_ expression cassette of the pMR plasmid (Table 2) and transformation yielded the recombinant *S. cerevisiae* Y294[EntA_Opt] strain.

### Antimicrobial Activity of Recombinant Strains

**Fig. 1:**
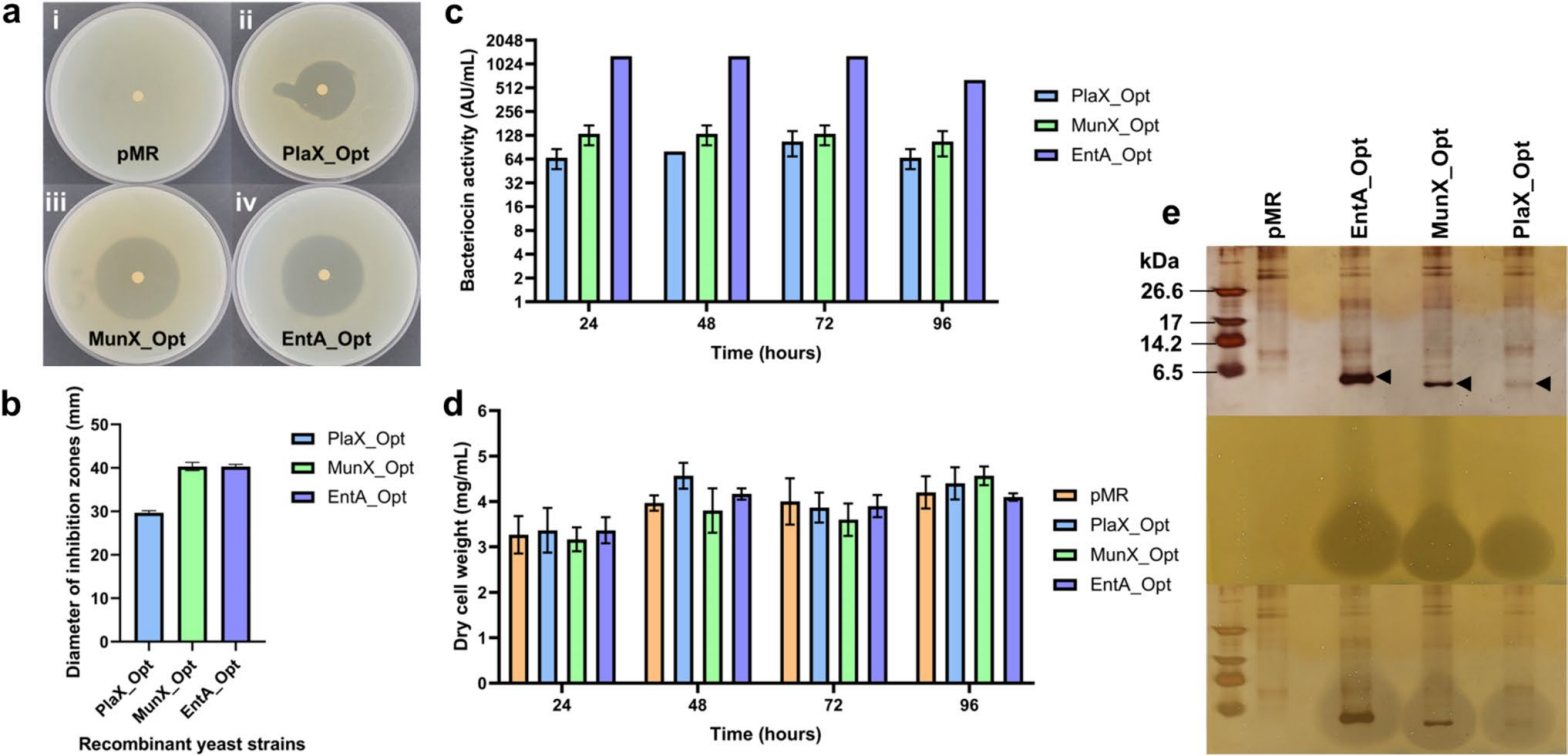
Antimicrobial activity of the recombinant *S. cerevisiae* Y294 strains against *L. monocytogenes* EDG-e, relative to the negative control, pMR. **a** Agar overlays of the yeast strains with *L. monocytogenes* and **b** the corresponding average diameter of inhibition zones. **c** Bacteriocin activity of the crude supernatant as determined with agar well-diffusion assays and **d** dry cell weight of the yeast strains. **e** Tricine SDS-PAGE analysis of 20-fold concentrated supernatant of EntA_Opt, MunX_Opt and PlaX_Opt. The panels include the silver-stained gel (top) with arrows indicating the recombinant peptides, the gel overlaid with *L. monocytogenes* (middle) and the superimposed gels (bottom).

The antimicrobial activity of the recombinant *S. cerevisiae* Y294[EntA_Opt] strain was evaluated against *L. monocytogenes* using agar overlay and agar well-diffusion assays and compared to the Y294[PlaX_Opt] and Y294[MunX_Opt] strains (Fig. 1). The inhibition zones on the overlays (yeast strains spotted onto agar plates and overlayed with *L. monocytogenes*) showed an average diameter of 40.33 ± 0.47 mm for Y294[EntA_Opt], compared to 29.66 ± 0.47 and 40.33 ± 0.94 mm for Y294[PlaX_Opt] and Y294[MunX_Opt], respectively (Fig. 1a and b). However, the well-diffusion assays (Fig. 1c) indicated 9.6-fold and 12-fold higher bacteriocin activity for EntA_Opt compared to MunX_Opt and PlaX_Opt after 72 h. A slight decrease in antimicrobial activity for all strains after 72 h could indicate cell lysis during the stationary phase, when proteolytic enzymes started to degrade the peptides. The dry cell weight (DCW) for the recombinant strains remained similar to the negative control strain, indicating that cell growth was unaffected by bacteriocin production (Fig. 1d). Tricine SDS-PAGE analysis of 20-fold concentrated supernatants from the recombinant strains indicated peptide bands corresponding to the expected size for enterocin A (EntA_Opt, 4.8 kDa), mundticin ST4SA (MunX_Opt, 4.2 kDa) and plantaricin 423 (PlaX_Opt, 3.9 kDa) (Fig. 1e). Corresponding peptides were absent in the negative control strain (pMR). Although SDS-PAGE is not quantitative, the peptide bands for enterocin were more intense than for mundticin and plantaricin. When the SDS-PAGE gels were overlaid with *L. monocytogenes*, clear inhibition zones corresponded to the respective peptides.

### Characterisation of Peptides Produced in Shake Flasks

Peptide production by Y294[EntA_Opt] was evaluated based on peptide concentration and MIC. The yeast was cultivated in shake flasks for 72 h, and the supernatant was harvested and purified with preparative HPLC. The eluted fractions were collected and spotted against *L. monocytogenes* EDG-e to identify the active peptide-containing fractions. Anti-listerial activity was identified in fractions D3 to G4, corresponding to the peaks observed at retention times 15.77 to 24.54 min on the HPLC chromatogram (Fig. S5).

All the active enterocin A fractions were combined, freeze-dried and analytically weighed to determine the concentration (Table 3) relative to recombinant plantaricin 423 and mundticin ST4SA [10]. The purity of the HPLC-purified EntA_Opt was assumed to be > 95% as determined by tricine SDS-PAGE analysis and densitometry (Fig. S6). The average concentration obtained for the HPLC-purified EntA_Opt was 50.93 ± 0.69 mg/L, more than twofold higher than PlaX_Opt and MunX_Opt. Notably, the MIC for EntA_Opt against *L. monocytogenes* EDG-e was 0.93 mg/L (192.74 nM), i.e. the yeast produced 54.76-fold more EntA_Opt than required to inhibit *L. monocytogenes*. However, the MICs for EntA_Opt and MunX_Opt were significantly lower than for PlaX_Opt, suggesting that PlaX_Opt is less active. A similar observation was reported for the expression of plantaricin 423 and mundticin ST4SA as His-tagged green fluorescent protein (GFP) fusions in *E. coli* [18]. Following HPLC purification, 121.29 mg/L of GFP-PlaX was produced, but conformational isomers of the liberated plantaricin 423 were observed, resulting in only 83.33 BU/mL, as opposed to 1 600 BU/mL for GFP-MunX.

**Table 3:** MIC and concentrations of purified peptides produced by Y294 strains

**Peptide**	**Concentration** **(mg/L ± SD)**	**MIC** **(mg/L ± SD)**	**Molecular mass (g/mol)**	**MIC (nM)**	**Reference**
EntA_Opt	50.93 ± 0.69	0.93 ± 0.0	4,825.26	192.74 ± 0.0	This study
PlaX_Opt	18.40 ± 3.40	4.65 ± 2.63	3,928.70	1183.67 ± 669.59	[10]
MunX_Opt	20.90 ± 1.59	0.46 ± 0.0	4,285.09	108.52 ± 0.0	[10]

*SD*, standard deviation

### LC-MS/MS Analysis of Post-Translational Modifications

**Fig. 2:**
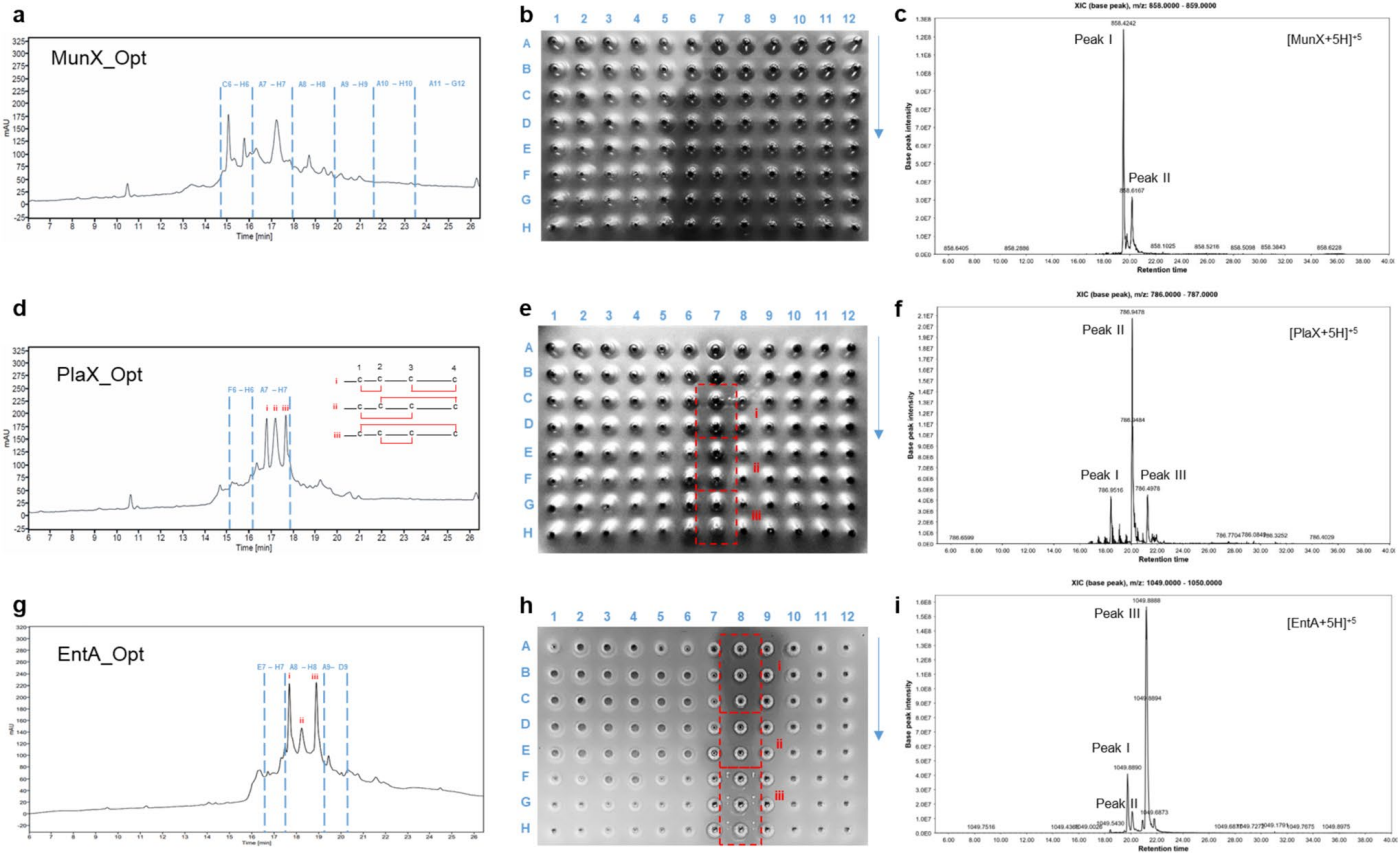
HPLC purification and LC-MS analysis of the bacteriocins. **a**, **d**, **g** HPLC fractionation of MunX_Opt, PlaX_Opt and EntA_Opt, respectively. **b, e, h** The corresponding anti-listerial activity of the collected fractions (spotted top to bottom, left to right). **c, f, i** LC-MS analysis with monoisotopic ions observed for each peptide carrying +5 charges [M+5]^+5^. Red boxes indicate the activity corresponding to the respective peaks.

**Fig. 3:**
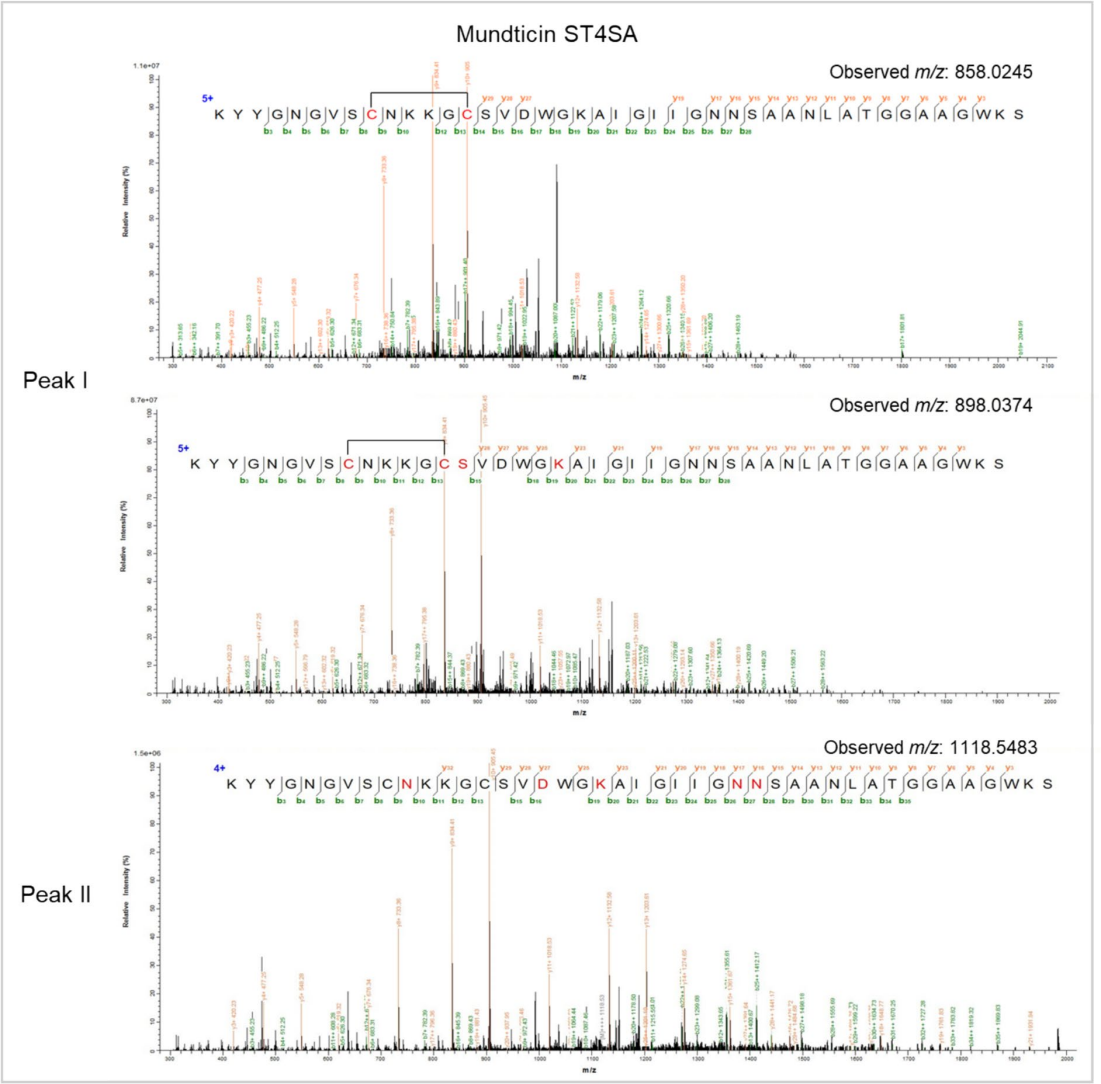
Tandem mass spectrometry of the monoisotopic parent ion from the mundticin ST4SA peptide envelopes observed at peak I (retention time 19.45–19.51 min) and peak II (retention time 20.17–20.19 min). Collision-induced peptide fragmentation spectra confirmed the MunX_Opt peptide sequence at peak I with one disulphide bond and at peak II with no disulphide bonds, with and without modifications. Adducts containing potential modifications such as deamination, succinylation, methylmalonylation and oxidation are indicated in red at the putative modification site

The HPLC-purified peptides from the C8 column were further resolved on a C18 analytical column (Fig. 2a, d, g), and the fractions tested for antimicrobial activity (Fig. 2b, e, h). The C6 - H12 fractions of MunX_Opt displayed anti-listerial activity and corresponded to the peaks at retention times 14.70 to 26.92 min (Fig. 2a, b). Nano-LC-MS/MS analysis of the active C18 fractions from the analytical column (Fig. 2c) showed two distinct peaks at retention times 19.45–19.51 and 20.17–20.19, corresponding to the monoisotopic ions carrying +5 charges [M+5]^+5^. The mass spectra of peak I at retention time 19.45 revealed isotopic envelopes of the [M+5H]^+5^ and [M+4H]^+4^ species, with an observed *m/z* 858.4253 and *m/z* 1072.7760, respectively (Table 4; Fig. S7). The accurate mass measurements closely agree with the theoretical monoisotopic mass of mundticin ST4SA without a disulphide bond (4287.0953 Da) (Fig. 3). At retention time 19.51 of peak I, isotopic envelopes of the [M-2H+3H]^+3^, [M-2H+4H]^+4^, [M-2H+5H]^+5^, [M-2H+6H]^+6^ species, corresponding to *m/z* 1429.3618, *m/z* 1072.2759, *m/z* 858.0245 and *m/z* 715.1857 were observed (Table 4; Fig. S8).

**Table 4:**
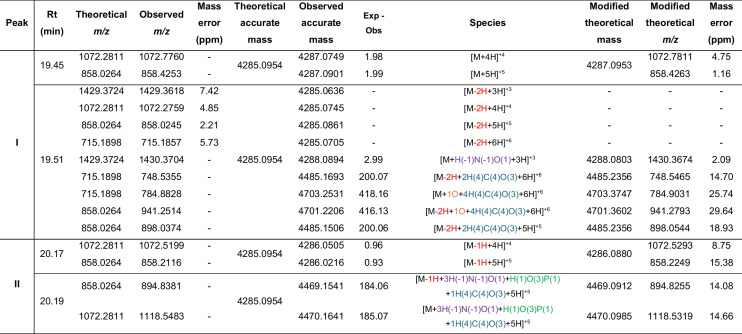
Recombinant mundticin ST4SA species identified with LC-MS/MS

Modifications indicated in red, hydrogens lost due to disulphide bond formation; light blue, succinyl or methylmalonylation; purple, deamination, orange indicates oxidation; green, phosphorylation

A higher abundance and greater intensity of the peptide species with the correct disulphide bonds were observed for MunX_Opt than species without disulphide bonds, indicating that the yeast was able to correctly fold most of the peptide species, resulting in active peptides. The terminology ‘peptide species’ refers to various molecular forms or isoforms of the expressed peptide, including the correctly folded, bioactive form and modified or misfolded variants. At peak I, peptide species containing the correct disulphide bonds (with and without modifications) were observed at a tenfold higher intensity than those lacking disulphide bonds (Fig. S8). At peak II, only species without disulphide bonds were observed.

These accurate mass measurements were in close agreement with the theoretical monoisotopic mass of mundticin ST4SA with a disulphide bond (4285.0954 Da), as two cysteine residues are oxidised, and two hydrogen atoms are lost. Furthermore, protein adducts of 200 Da to the mass of mundticin ST4SA were observed (*m/z* 898.0374 and *m/z* 748.5355). These measurements correspond to the addition of succinyl groups on a lysine (K) residue and methylmalonylation of a serine (S) residue within the amino acid sequence of the peptide, as putatively confirmed with tandem mass spectrometry (MS/MS) analysis (Fig. 3; Fig. S9). The species corresponding to the monoisotopic masses of these adducts are [M-2H+2H(4)C(4)O(3)+5H]^+5^ and [M-2H+2H(4)C(4)O(3)+6H]^+6^, respectively. Moreover, adducts of an additional 400 Da were observed, as well as oxidation of the peptide species (Table 4). The mass spectra of peak II at retention times 20.17–20.19 revealed envelopes of the [M-H+5H]^+5^ and [M-H+4H]^+4^ species, with an observed *m/z* 858.2116 and *m/z* 1072.5199, respectively (Table 4; Fig. S10). The accurate mass measurements correspond to the loss of one or two hydrogen atoms as one or no cysteine residues are oxidised (Table 4; Fig. 3; Fig. S11). Additional modifications, including deamination, phosphorylation and succinylation, were observed with the MS/MS analysis.

The HPLC-purified MunX_Opt was analysed with GC-MS to determine the potential conjugation of fatty acids to the peptide species (Fig. S12). Palmitic acid (C16-fatty acid) and stearic acid (C18-fatty acid) were detected in the highest abundance (18.658 ppm and 16.095 ppm, respectively). Myristic acid (C14), vaccenic acid (C18:1), arachidic acid (C20), dihomo-γ-linolenic acid (C20:3n6) and lignoceric acid (C24) were at lower abundance. From the HPLC chromatogram in Fig. 2a, multiple active peaks were observed for MunX_Opt over an extended retention time. This could result from fatty acids bound to the peptide, which alter the hydrophobicity, size, and structure of the peptide. Consequently, peptide species containing different fatty acids elute at different times. Further analysis would be required to determine the impact of each conjugated fatty acid as they could reduce or enhance activity.

Fractions F6 to H7 of the purified PlaX_Opt displayed anti-listerial activity and corresponded to the peaks at 15.39 to 17.92 min on the chromatogram (Fig. 2d and e). Three distinct peaks with similar intensity were observed, with peak I corresponding to active fractions C7 and D7, peak II to active fractions E7 and F7, and peak III to active fractions G7 and H7. The activity of the three distinct peaks varies, with the largest inhibition zones observed for peak I, followed by peak II and III. Since plantaricin 423 contains four cysteine residues, two disulphide bonds are formed, i.e. Cys1–Cys2 and Cys3–Cys4. However, two other conformations are theoretically possible, i.e. Cys1–Cys3 and Cys2–Cys4; or Cys1–Cys4 and Cys2–Cys3. The disulphide bond conformations affect the structure and hydrophobicity of the peptide species, causing them to elute at different times from the analytical column. It is hypothesised that peaks I, II and III for plantaricin 423 represent the three different disulphide bond conformations and that the peak with the highest activity represents the most active conformation.

The active fractions from the C18 analytical column were combined and analysed with nano-LC-MS/MS (Fig. 2f). Three distinct peaks were observed at retention times 18.40 - 18.42 min, 20.01 - 20.06 min, and 20.35 - 21.27 min, corresponding to the monoisotopic ions carrying +5 charges [M+5]^+5^. The mass spectra of peak I at 18.40 min revealed isotopic envelopes of the [M-4H+3H]^+3^, [M-4H+4H]^+4^, [M-4H+5H]^+5^ and [M-4H+6H]^+6^ species, with an observed *m/z* 1310.5787, *m/z* 983.1856, *m/z* 786.7479 and *m/z* 655.7916, respectively (Table 5; Fig. S13). The accurate mass measurements closely agree with the theoretical monoisotopic mass of plantaricin 423 with two disulphide bonds (3928.7101 Da).

**Fig. 4:**
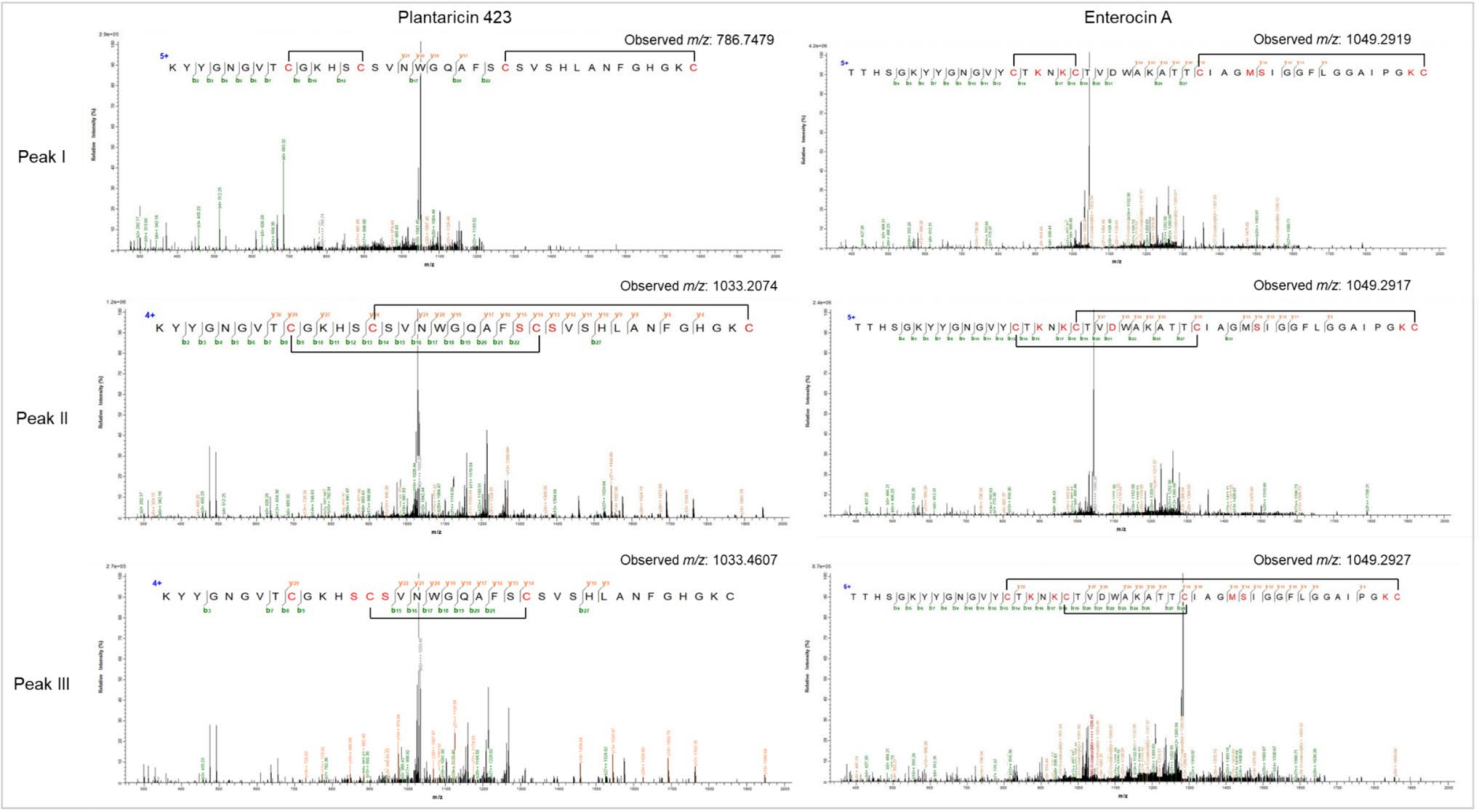
Tandem mass spectrometry of the monoisotopic parent ion from the peptide envelopes observed at the three peaks for PlaX_Opt and EntA_Opt, respectively. Collision-induced peptide fragmentation spectra putatively confirmed the peptide sequences with disulphide bonds formed between Cys1–Cys2 and Cys3–Cys4 at peak I, Cys1–Cys3 and Cys2–Cys4 at peak II, and Cys1–Cys4 and Cys2–Cys3 at peak III

The putative disulphide bond conformations were determined with MS/MS analysis (Fig. 4). The disulphide bonds of the peptide species observed at peak I are likely the Cys1–Cys2 and Cys3–Cys4 conformation, as observed by the fractionation of the ions. Peak I also has the highest antimicrobial activity, yielding the largest inhibition zones (Fig. 2e). Furthermore, at retention time 18.42 of peak I, an isotopic envelope of the [M-2H+2H(4)C(4)O(3)+5H]^+5^ species corresponding to *m/z* 827.1673 was observed, with its accurate mass measurement closely agreeing with the theoretical monoisotopic mass of plantaricin 423 containing only one disulphide bond with a 200 Da adduct (4130.8001 Da). The MS/MS analysis revealed a Cys1-Cys2 disulphide bond and that the serine residues at sites 13 and 15 underwent methylmalonylation (Fig. S14).

The mass spectra of peak II at 20.01 and 20.06 min revealed envelopes of plantaricin 423 species with two, three, four or no oxidised cysteine residues (Table 5; Fig. S15). However, the species with four oxidised cysteine residues containing two disulphide bonds) were the most abundant, with their intensity on the mass spectra at least 22-fold higher than those with one or no disulphide bonds. However, based on the fractionation spectra of the MS/MS analysis, the potential disulphide bonds are Cys1–Cys3 and Cys2–Cys4 (Fig. 4). In addition, 200 Da and 400 Da adducts were observed for the peptide species, indicating possible methylmalonylation of the serine residues (Fig. S16). The mass spectra of peak III at retention times 20.35 to 21.27 min also revealed species with one, two, three or four oxidised cysteine residues (Fig. S17). The species containing adducts of 200 Da and 400 Da were the most abundant. The MS/MS analysis indicated potential Cys1–Cys4 and Cys2–Cys4 disulphide bond conformations (Fig. S18).

**Table 5:**
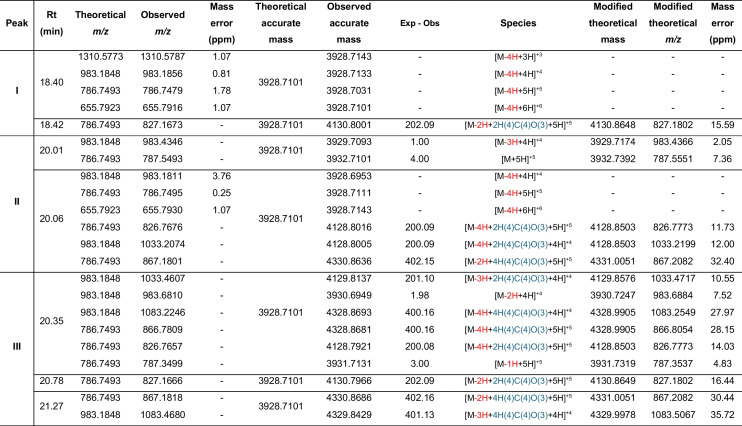
Recombinant plantaricin 423 species identified with LC-MS/MS

Modifications indicated in red, hydrogens lost due to disulphide bond formation; light blue, succinyl or methylmalonylation

The different disulphide bond conformations and post-translational modifications impact the 3D structure of the peptide and a change in hydrophobicity, causing the different peptide species to elute at different retention times. At peak I, most of the peptide species contain no modifications, whereas the number of modifications and peptide size increase, and they become more hydrophobic at peak II and peak III. A higher hydrophobicity results in a stronger interaction with and retention on the C18 column. Species without modifications that still contain two disulphide bonds eluted at different times, and the fractionation spectra were different for each peptide. This suggests different disulphide bond conformations that alter the peptide structures and change their hydrophobicity. This also substantiates the observation that modified peptide species with similar monoisotopic masses eluted at different retention times (Table 5). Furthermore, the antimicrobial activity decreased from peak I to peak III, potentially resulting from these modifications.

Fractions E7 to D9 of the purified EntA_Opt displayed anti-listerial activity and corresponded to the peaks at 16.60–20.17 min on the chromatogram (Fig. 2g and h). Three distinct peaks with different intensities were observed on the chromatogram, with peak I corresponding to the active fractions A8 – C8, peak II corresponding to active fractions D8 – E8 and peak III corresponding to active fractions F8 - H8. The activity of peak I was the highest (and showed the largest inhibition zone), followed by peak III and peak II. Enterocin A contains four cysteine residues, i.e. two disulphide bonds are formed. However, only two peaks with similar intensity were observed, suggesting that more peptide species with the correct disulphide bond conformation were produced relative to plantaricin 423 that had three peaks with similar intensity.

The active fractions of EntA_Opt following C18 purification were combined and analysed with nano-LC-MS/MS (Fig. 2i). Three distinct peaks were observed at retention times 19.65–19.74, 20.09 and 21.19, corresponding to the monoisotopic ions carrying +5 charges [M+5]^+5^. The mass spectra of peak I revealed isotopic envelopes of enterocin A peptide species that contain mainly two disulphide bonds (four oxidised cysteine residues), but also species where one, two or no cysteines were oxidised (Table 6; Fig. S19; Fig. S20). The disulphide bonds of the peptide species observed at peak I are hypothesised to be the Cys1–Cys2 and Cys3–Cys4 conformation as observed by the fractionation of the ions. Peak I also resulted in the largest inhibition zones (Fig. 2h). All the identified species were oxidised with a varying number of oxygen atoms. The MS/MS results showed that oxidation occurred on the methionine residue (Fig. 4; Fig. S21), whereas succinylation and methylmalonylation occurred on the lysine and serine residues, respectively. Furthermore, species of *m/z* 969.6609, *m/z* 808.2189 and *m/z* 1211.8202 were deaminated on an asparagine residue in addition to oxidation on the methionine residue. These accurate mass measurements closely agree with the theoretical monoisotopic mass of enterocin A with two disulphide bonds (4843.2295 Da).

**Table 6:**
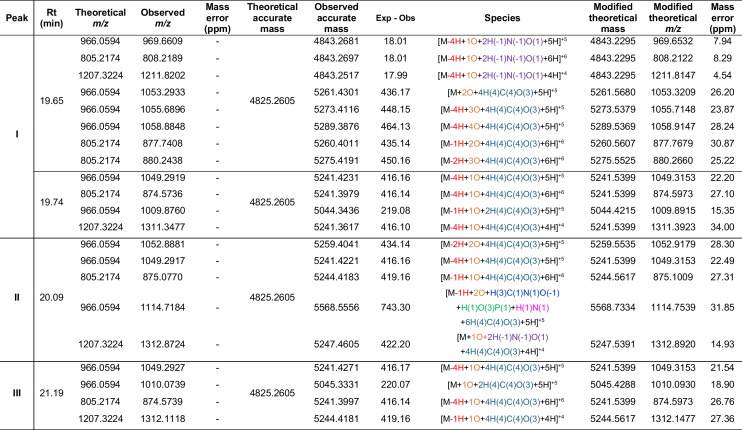
Recombinant enterocin A species identified with LC-MS/MS

Modifications indicated in red, hydrogens lost due to disulphide bond formation; light blue, succinyl or methylmalonylation; purple, deamination; orange, oxidation; green phosphorylation; pink, amino groups; dark blue, methylamine groups

At peak II (retention time 20.09 min), only one potentially active species (containing two disulphide bonds) [M-4H+1O+4H(4)C(4)O(3)+5H]^+5^ was identified, which corresponded to *m/z* 1049.2917 (Table 6; Fig. S22). The MS/MS analysis revealed that the disulphide bond was formed between Cys1–Cys3 and Cys2–Cys4 (Fig. 4). Peptide species containing one or no disulphide bonds were abundant; one of the most abundant species (*m/z* 1114.7184) contained additional modifications, which included succinylation, methylmalonysation, phosphorylation, methylamination, oxidation and the addition of an amino group as determined by MS/MS analysis (Fig. S23). The lower activity observed for this peak (Fig. 2h) could result from the high abundance of peptide species containing no or single disulphide bonds, as well as the additional modifications.

At peak III (retention time 21.19 min), peptide species with four, one or no oxidised cysteine residues were detected (Table 6; Fig. S24). All species contained modifications of succinylation on lysine residues, methylmalonysation on serine residues and oxidation on methionine residues (Fig. 4, Fig. S25). However, the fractionation spectra from the MS/MS analysis indicated potential disulphide bonds forming between Cys1–Cys4 and Cys2–Cys3 (Fig. 4).

Similar to the LC-MS/MS results of plantaricin 423, different conformations of disulphide bonds were detected for enterocin A, as well as the addition of various post-translational modifications. This resulted in the different enterocin A species eluting at different times: the Cys1–Cys2 and Cys3–Cys4 species eluting at peak I; Cys1–Cys3 and Cys2–Cys4 species eluting at peak II and Cys1–Cys4 and Cys2–Cys3 species eluting at peak III. The more modifications the peptide species underwent, and the more disulphide bonds were reduced, the more the peptide activity declined, as seen with the species detected at peak II. This was also evident for plantaricin 423 as the species at peaks II and III contained more modifications and displayed lower activity.

Although different conformations of disulphide bonds were produced for enterocin A, most of the peptide species contained two disulphide bonds with either Cys1–Cys2 and Cys3–Cys4 or Cys1–Cys4 and Cys2–Cys3 conformations. In contrast, the plantaricin 423 peptide species contained three disulphide bond conformations in equal abundance. In addition, more plantaricin species were detected with reduced disulphide bonds compared to enterocin, which confirms the lower activity observed for plantaricin with the MICs. Nevertheless, a significant amount of incorrectly folded and inactive peptides was produced, which could complicate the downstream purification of active peptides.

### Peptide Production in Bioreactors

**Fig. 5:**
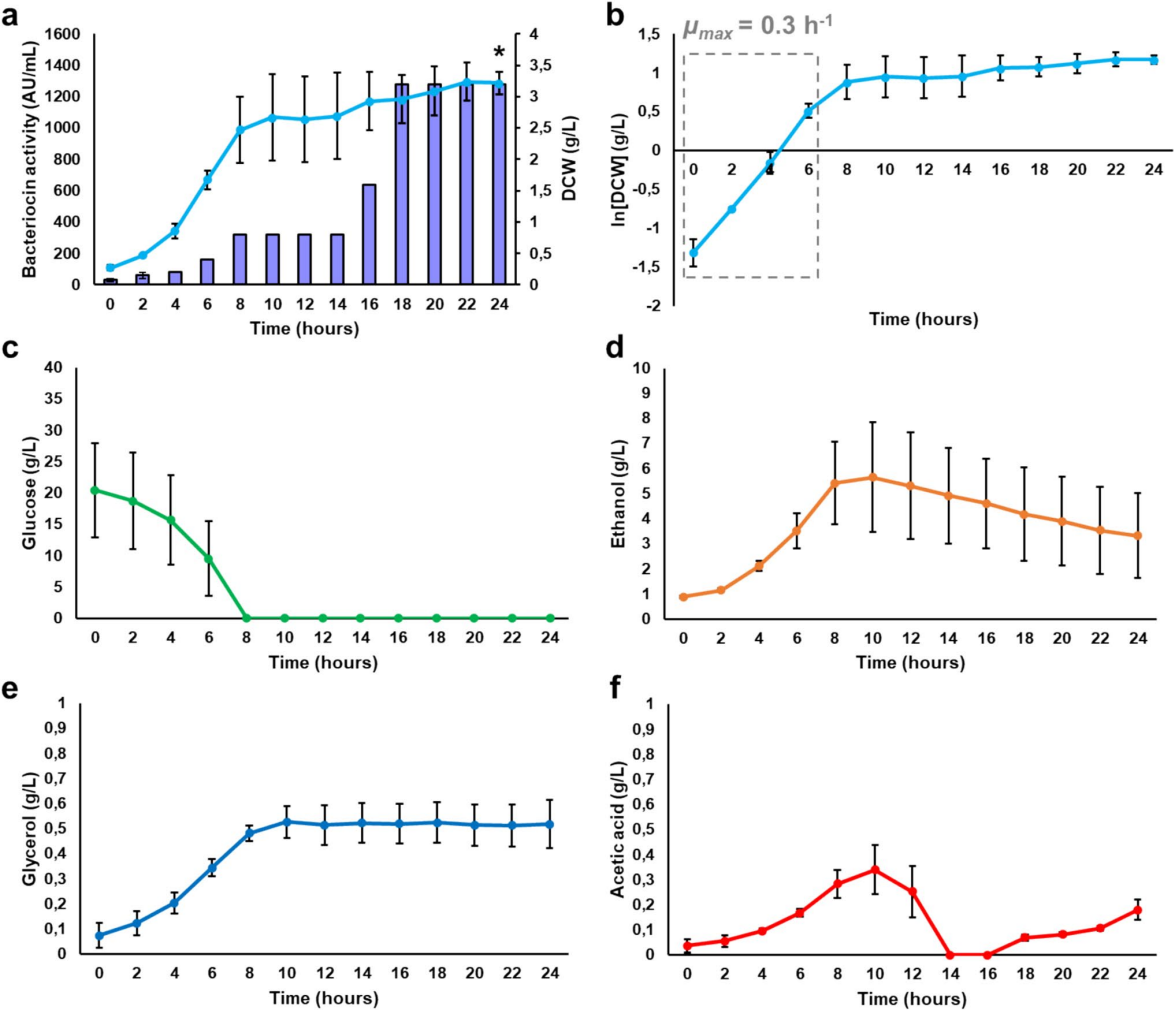
Batch cultivation of Y294[EntA_Opt] to produce enterocin A, showing **a** extracellular bacteriocin activity (purple columns) and DCW (blue line), and **b** maximal specific growth rate (*µ*_max_*)* during exponential growth. The supernatant was harvested at 24 h, and **c** glucose, **d** ethanol, **e** glycerol and **f** acetic acid were quantified with HPLC analysis.

The Y294[EntA_Opt] strain produced the most peptide in shake flasks and was selected for up-scaled peptide production in bioreactors under batch and fed-batch cultivation conditions. Peptide production was first evaluated under batch cultivation conditions for 24 h, with DCW, bacteriocin activity, glucose consumption and ethanol, glycerol and acetic acid production monitored (Fig. 5). The pH and temperature were maintained at 5.5 and 30 °C, while a constant flow of air was provided. The exponential growth phase lasted about 6 h (Fig. 5a), when the extracellular bacteriocin activity reached 320 ± 0.0 AU/mL with DCW at 2.47 ± 0.88 g/L. However, maximal peptide activity (1 280 AU/mL) was observed at 18 to 24 h, when DCW reached 3.22 ± 1.17 g/L. The maximal specific growth rate (*µ*_max_ = 0.3 h^−1^) represents the slope of the natural logarithm of the DCW plotted as a function of time of the exponential phase (Fig. 5b; Fig. S26). The yeast consumed all the glucose (20 g/L) in the growth medium within the first 8 h (Fig. 5c). The biomass yield (*Y*_*X/S*_) at the end of the exponential phase was 0.07 g/g glucose, with an average of 0.18 g/g glucose after 24 h.

The Y294[EntA_Opt] strain produced 5.65 ± 2.18 g/L ethanol at the end of the exponential phase (Fig. 5d), but once the glucose was depleted, it used the ethanol as a carbon source, with only 3.31 ± 1.69 g/L ethanol left after 24 h. The DCW slowly increased from 14 h after a plateau between 8 and 14 h, suggesting some level of diauxic growth as the yeast cells started to consume ethanol (Fig. 5a). Low concentrations of glycerol (Fig. 5e) and acetic acid (Fig. 5f) were produced, reaching 0.53 ± 0.06 g/L and 0.34 ± 0.10 g/L, respectively. The acetic acid concentration decreased significantly as the yeast entered the stationary phase, suggesting that this served as a carbon source when glucose was depleted. It is essential to note that the bioreactor studies were conducted in duplicate, with relatively large standard deviations due to high variability typically associated with bioreactor studies. However, it was important to determine the trend for carbon conversion that informed the glucose feed for subsequent fed-batch cultivations.

**Fig. 6:**
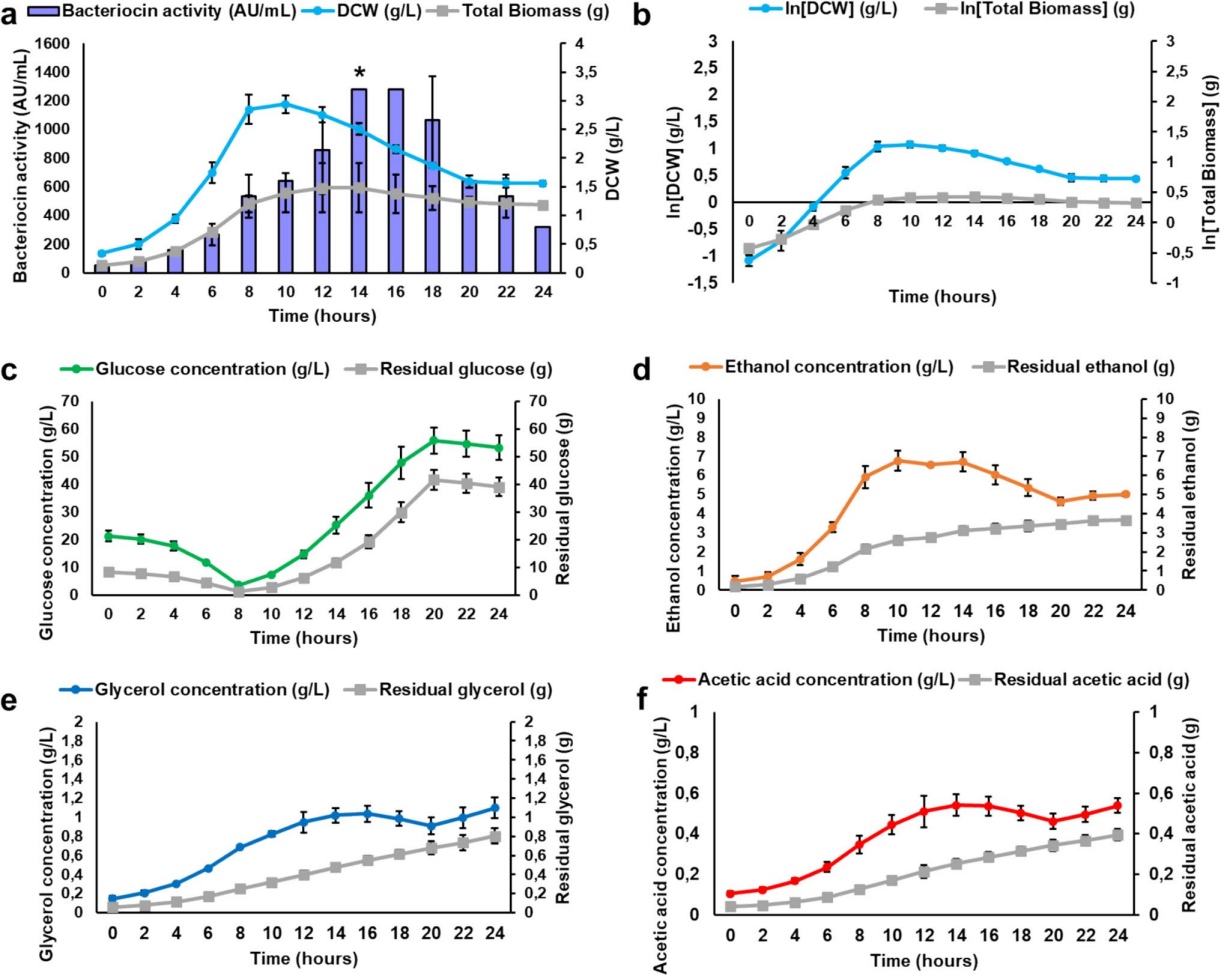
Fed-batch cultivation of Y294[EntA_Opt] for enterocin A production, with **a** extracellular bacteriocin activity and DCW monitored, and **b** the maximal specific growth rate (*µ*_max_*)*. The supernatant was harvested at 24 h, and **c** glucose, **d** ethanol, **e** glycerol and **f** acetic acid were quantified by HPLC analysis. The residual metabolite concentrations are indicated in grey, considering the dilution effect due to the substrate feed.

For the fed-batch cultivation of the Y294[MFα1_EntA_Opt] strain, glucose was fed upon the depletion of the initial carbon source to retain the yeast in the exponential phase. This can increase biomass production, resulting in increased production of recombinant peptides. However, the substrate feed rate must be regulated to ensure that the residual substrate concentration is maintained below the catabolite repression threshold. The maximal specific growth rate and biomass yield determined for the exponential growth phase of the batch cultivation were used to determine the flow rate (F) for the glucose feed for the fed-batch cultivation. The parameters for the fed-batch cultivation are summarised in Tables S2 and S3. The selected growth rate was 50% of *µ*_max_ (initial volume of 400 mL), and stepwise exponential glucose feed was applied upon glucose depletion at 8 h. The flow rate of the glucose feed is depicted in Fig. S26, with all the parameters for the fed-batch model summarised in Fig. S27. Peptide production was again evaluated for 24 h, and the DCW, bacteriocin activity, glucose consumption and ethanol, glycerol and acetic acid production were monitored (Fig. 6). The pH and temperature were maintained at 5.5 and 30 °C while a constant flow of air was provided.

Exponential growth was observed for the first 10 h (Fig. 6a), when the extracellular bacteriocin activity reached 640 ± 0.0 AU/mL and the DCW was 2.94 ± 0.16 g/L. The exponential glucose feed was implemented at 8 h (after the initial batch phase), but the DCW reduced significantly after 10 h. The increase in media volume due to the substrate feed was taken into account, and the total biomass was calculated (Fig. 6a). The total biomass reached 1.48 ± 0.43 g at 12 h and remained stationary [as confirmed by the natural logarithm of the biomass (Fig. 6b)], but decreasing slightly to 1.19 ± 0.04 g after 24 h. Maximal peptide activity (1 280 ± 0.0 AU/mL) was observed at 14 h, but decreased significantly after 18 h, with only 320 ± 0.0 AU/mL left after 24 h. Maximal peptide production correlated with a biomass yield (*Y*_*X/S*_) of 0.08 g biomass/g glucose. The decreased peptide activity could be linked to the decrease in biomass, as well as possible cell lysis during the stationary phase, which releases proteases that degrade the peptides.

Glucose accumulated in the fermentation broth when the glucose feed was implemented after the initial batch phase (Fig. 6c). This could have resulted in osmotic stress and declining DCW as the high levels of glucose inhibit cell growth. Light microscopy (Fig. S28) showed that the yeast cells started to aggregate and form clumps after 10 h, suggesting a potential stress response. Higher concentrations of ethanol (6.77 ± 0.54 g/L) were recorded relative to batch cultivation (Fig. 6d), but it was less than 1% of the total volume, indicating that the yeast metabolism remained in the respiratory phase and the Crabtree effect was not initiated. Although slightly higher levels of glycerol and acetic acid were produced during fed-batch cultivation than batch cultivation (Fig. 6e and f), they were too low to impact yeast growth.

To determine the peptide yields, supernatant from the batch and fed-batch cultivations was harvested when maximal peptide activity was observed (Table 7). The purity of the peptides was confirmed with tricine-SDS-PAGE analysis and densitometry (Fig. S29). The Y294[EntA_Opt] strain yielded 50.93 ± 0.69 mg/L peptide in shake flasks, whereas the peptide yields increased to 77.22 ± 2.29 mg/L in batch and 85.77 ± 2.77 mg/L in fed-batch, representing a 52% and 68% improvement over shake flasks, respectively. However, batch cultivation allowed for more peptide production per substrate, with 4.32 ± 1.47 mg of peptide per gram of glucose. In contrast, the fed-batch only yielded 2.69 ± 0.06 mg/g glucose. Although the maximum peptide levels were reached faster (14 h) than with batch cultivation (18–24 h), fed-batch cultivation required more glucose, which makes the production process more expensive. Nevertheless, the productivity obtained in the bioreactors was significantly higher than with shake flasks, with the fed-batch cultivation proving to be the most productive, as more peptide was produced in a shorter time.

**Table 7:** Peptide yields from Y294[EntA_Opt] under different culture conditions

	**Peptide concentration (mg/L) ± SD**	**Peptide yield (mg peptide/g glucose consumed) ± SD**	**Biomass yield (*Y***_***x*/s**_)* (g biomass/g glucose) **± SD**	**Time harvested**	**Peptide productivity (mg/L/h)**
**Shake flasks**	50.93 ± 0.69	2.55 ± 0.03	0.19 ± 0.01	72 h	0.71
**Bioreactors**					
Batch cultivation	77.22 ± 2.29	4.32 ± 1.47	0.18 ± 0.06	24 h	3.22
Fed-batch cultivation	85.77 ± 2.77	2.69 ± 0.06	0.08 ± 0.00	14 h	6.13

*Calculated when maximal peptide activity was observed, and the supernatant was harvested

*SD*, standard deviation

## Discussion

Following previous success with the recombinant expression of plantaricin 423 and mundticin ST4SA [10], this study investigated the expression of the class IIa enterocin A (native to *E. faecium*) in the *S. cerevisiae* Y294 laboratory strain. Enterocin A was previously expressed in the yeasts *P. pastoris*, *K. lactis* and *H. polymorpha* [17], but the low CBI of the native enterocin A gene (*entA*) suggested that it would not be easily expressed in *S. cerevisiae*. Codon optimisation of the mature enterocin A increased the CBI from 0.07 to 0.48, and the *entA_Opt* gene was cloned in-frame with the MFα1 secretion signal under the control of the *ENO1* promoter and terminator. Following yeast-mediated ligation, the Y294[EntA_Opt] strain was evaluated for peptide production and antimicrobial activity against *L. monocytogenes* as indicator organism. The results were compared to the strains producing plantaricin 423 and mundticin ST4SA, i.e. Y294[PlaX_Opt] and Y294[MunX_Opt] [10].

The results confirmed the successful expression of enterocin A in *S. cerevisiae*. The bacteriocin activity of the Y294[EntA_Opt] strain was 9.6-fold higher than Y294[MunX_Opt] and 12-fold higher than Y294[PlaX_Opt] after 72 h. There was no significant difference in DCW of the recombinant and control strains, indicating that bacteriocin production did not impact cell growth. The MIC of EntA_Opt against *L. monocytogenes* EDG-e was 192.74 nM, compared to 1183.67 nM for PlaX_Opt and 108.52 nM for MunX_Opt. The average yield of EntA_Opt in small-scale shake flasks was 50.93 mg/L, 2.77-fold higher than PlaX_Opt and 2.44-fold higher than MunX_Opt. The intensity of the recombinant peptide bands with Tricine-SDS-PAGE analysis also suggested that more EntA_Opt was produced than MunX_Opt and PlaX_Opt.

The higher activity observed for EntA_Opt could be ascribed to more enterocin peptides being produced in *S. cerevisiae*, but this did not explain the much higher MIC for PlaX_Opt against *L. monocytogenes*. An important consideration for expression in yeast is effective disulphide bond formation: both EntA_Opt and PlaX_Opt require two disulphide bonds for activity, yet the MIC of PlaX_Opt was 6.14-fold higher than for EntA_Opt. In contrast, the MIC of EntA_Opt was only 1.78-fold higher than MunX_Opt, which has only one disulphide bond. The LC-MS/MS analysis confirmed that more peptides with the correct disulphide bond conformation were produced for enterocin A than plantaricin 423, explaining the higher specific activity.

A disulphide bond accessory protein, PlaC, is expressed from the native plantaricin 423 operon as a chaperone for correct disulphide bond formation [29]. Since only the mature peptides were expressed in *S. cerevisiae* Y294, only the native *S. cerevisiae* protein disulphide isomerase (PDI) is available to assist with disulphide bond formation [30]. The absence of the PlaC accessory protein could result in peptides with various disulphide bond conformations and less activity. The co-expression of the *plaC* gene in *S. cerevisiae* Y294[PlaX_Opt] should thus be investigated to determine if more peptide species with the correct conformation of disulphide bonds will be produced, thus resulting in a higher specific activity for PlaX_Opt. Similarly, the over-expression of the *PDI1* gene in the recombinant yeast strains could be explored, in particular for mundticin ST4SA and enterocin A that do not have accessory proteins for disulphide bonds in their operons.

In addition to different disulphide bond conformations, the activity of the recombinant peptides may be affected by various post-translational modifications as observed by LC-MS/MS analysis. The antimicrobial activity of a peptide is correlated with the peptide’s overall amphipathicity, hydrophobicity and flexibility [31]. Grimsey et al. [32] observed an increase in antimicrobial activity for two peptides upon the incorporation of shorter-chain fatty acids (C8 and C10) and a decrease in activity for longer-chain fatty acids (C12 and C14). However, Makovitzki et al. [33] found that incorporating palmitic acid (C16) rendered the most active form of several different peptides. Furthermore, the incorporation of fatty acids could also improve the stability and shelf-life of peptides [34]. In this study, various fatty acids were associated with MunX_Opt, which could impact the activity of the peptide. However, a follow-up study should investigate the antimicrobial activity of each fatty acid-conjugated peptide species.

Other post-translational modifications detected included succinylation, methylmalonylation, deamination, oxidation, phosphorylation, and the addition of amino or methylamine groups, which alter the peptide’s hydrophobicity and can impact its antimicrobial activity. For example, oxidation of methionine residues typically occurs during the purification of peptides or long-term storage, which can alter the polarity of the amino acid residue and the structure and subsequent function of a protein or peptide [35, 36]. Although Johnsen et al. [37] reported that the specific activity of oxidised pediocin PA-1 was 100-fold lower than the unoxidised form, Goumon et al. [38] showed that the oxidation state of the methionine residue is not essential for the antimicrobial activity of the proenkephalin-A peptide.

Despite the various post-translational modifications of the peptides, the production levels and peptide activity of recombinant enterocin A reported in this study remain unparalleled. The production of enterocin A by *S. cerevisiae* Y294[EntA_Opt] was subsequently evaluated in bioreactors, which allow for more reproducible, high-yielding recombinant protein production that can be further optimised by regulating the growth conditions via real-time monitoring. During fermentation, high ethanol concentrations can suppress cell growth, and glucose is directed to ethanol rather than biomass production (the Crabtree effect [39]), resulting in low recombinant protein yields. This can be avoided by maintaining a high dissolved oxygen level, which ensures oxidative metabolism and results in improved biomass and recombinant peptide production.

During batch cultivation, the Y294[EntA_Opt] strain yielded 77.22 ± 2.29 mg/L at 18–24 h (Table 7). During fed-batch cultivation, fresh glucose was fed into the bioreactor upon depletion of the initial glucose. Although fed-batch cultivation allows the yeast to maintain exponential growth for longer, high residual glucose concentrations in the growth media coupled with a high specific growth rate may promote catabolite repression in addition to the Crabtree effect. Therefore, the growth rate during fed-batch cultivation was maintained at 50% of the maximum growth rate obtained during the exponential phase of batch cultivation. The yeast grew exponentially for 8 h, entered the stationary phase and achieved maximal peptide production (85.77 ± 2.77 mg/L) at 14 h. Compared to the shake flasks, the peptide yields improved 1.52-fold during batch and 1.68-fold during fed-batch cultivation in the bioreactors. Although higher yields were obtained for fed-batch cultures, they required larger quantities of glucose, thereby increasing costs compared to batch cultures. The batch fermentation demonstrated greater efficiency for peptide production, producing 4.31 mg peptide/g glucose, compared to 2.69 mg peptide/g glucose during fed-batch cultivation.

The yields obtained for enterocin A from shake flasks and bioreactors represented a significant improvement over yields previously reported for plantaricin 423 and mundticin ST4SA [10]. It also outperformed previous studies on heterologously expressed bacteriocins. For example, GFP-MunX expressed in *E. coli* yielded 153.30 mg/L culture, resulting in 12.4 mg/L active mundticin ST4SA after liberation and HPLC purification. Although GFP-PlaX was produced at 121.29 mg/L culture, evidence suggested that conformational isomers were responsible for a relatively low specific activity. Furthermore, the recombinant enterocin A produced in *S. cerevisiae* achieved nearly a twofold higher yield than reported by Borrero et al. [17], who obtained 45.1 mg/L enterocin A in *P. pastoris*. The yeast expression system reported here thus represents a promising method for producing recombinant enterocin A.

## Conclusions

In this study, codon-optimised enterocin A was successfully expressed in the *S. cerevisiae* Y294 laboratory strain and significantly higher yields of the recombinant peptide were obtained in shake flasks than previously reported for plantaricin 423 and mundticin ST4SA [10]. The antimicrobial activity of plantaricin 423 was sixfold lower than enterocin A and tenfold lower than mundticin ST4SA, which could be a result of more peptides with the wrong disulphide bond conformations due to the absence of the PlaC accessory protein that facilitates disulphide bond formation in the native host. In addition, LC-MS/MS analysis showed various post-translational modifications of the peptides that could affect the specific activity of the peptide. Furthermore, peptide yields by the *S. cerevisiae* Y294 strain-producing enterocin A improved 1.52-fold with batch and 1.68-fold with fed-batch cultivation in bioreactors, compared to growth in shake flasks. However, the batch cultivation was more efficient as more peptide was produced per gram of carbon input.

This study demonstrated that *S. cerevisiae* is a promising host to produce class IIa bacteriocins, but a significant amount of incorrectly folded and inactive peptides was produced that could complicate downstream purification of active peptides. This could be addressed by investigating the co-expression of disulphide bond accessory proteins to facilitate correct disulphide bond formation. Comparing MICs of recombinant peptides from yeast (with and without disulphide bond accessory proteins) to those of native hosts would also allow for better comparison of specific activity. However, this would require the same level of purity to accurately compare the activity levels, and purification of bacteriocins from their native hosts remains a bottleneck. Moreover, the production of enterocin A in bioreactors can be further optimised by evaluating different growth parameters, carbon sources and feedstocks, including industrial waste products as carbon sources for more sustainable peptide production. Furthermore, future studies should investigate the effect of individual post-translational modification on the activity of the peptide species, as well as the characterisation of the fatty acid-conjugated peptide species.

## Supplementary Information

Below is the link to the electronic supplementary material.

Supplementary file1 (DOCX 15780 KB)

## Data Availability

No datasets were generated or analysed during the current study.

## Declarations

### Disclaimer

Opinions communicated and all conclusions arrived at are those of the authors and are not necessarily to be attributed to the NRF.

## References

[1] Darbandi A, Asadi A, Mahdizade Ari M, Ohadi E, Talebi M, Halaj Zadeh M, Emamie AD, Ghanavati R, Kakanj M (2022) Bacteriocins: properties and potential use as antimicrobials. J Clin Lab Anal 36(1):e24093. 10.1002/jcla.24093 34851542 10.1002/jcla.24093 PMC8761470

[2] Lozo J, Topisirovic L, Kojic M (2021) Natural bacterial isolates as an inexhaustible source of new bacteriocins. Appl Microbiol Biotechnol 105(2):477–492. 10.1007/s00253-020-11063-3 33394148 10.1007/s00253-020-11063-3

[3] Yi Y, Li P, Zhao F, Zhang T, Shan Y, Wang X, Liu B, Chen Y, Zhao X, Lü X (2022) Current status and potentiality of class II bacteriocins from lactic acid bacteria: structure, mode of action and applications in the food industry. Trends Food Sci Technol 120:387–401. 10.1016/j.tifs.2022.01.018

[4] Lohans CT, Vederas JC (2012) Development of class IIa bacteriocins as therapeutic agents. Int J Microbiol 2012:1–13. 10.1155/2012/386410 10.1155/2012/386410 PMC3236453 22187559

[5] Chikindas ML, Weeks R, Drider D, Chistyakov VA, Dicks LMT (2018) Functions and emerging applications of bacteriocins. Curr Opin Biotechnol 49:23–28. 10.1016/j.copbio.2017.07.011 28787641 10.1016/j.copbio.2017.07.011 PMC5799035

[6] Mathur H, Beresford TP, Cotter PD (2020) Health benefits of lactic acid bacteria (LAB) fermentates. Nutrients 12(6):1679. 10.3390/nu12061679 32512787 10.3390/nu12061679 PMC7352953

[7] Barbiroli A, Musatti A, Capretti G, Iametti S, Rollini M (2017) Sakacin-A antimicrobial packaging for decreasing *Listeria* contamination in thin-cut meat: preliminary assessment. J Sci Food Agric 97:1042–1047. 10.1002/jsfa.8120 27790709 10.1002/jsfa.8120 PMC5324655

[8] Guitián MV, Ibarguren C, Soria MC, Hovanyecz P, Banchio C, Audisio MC (2019) Anti-*Listeria monocytogenes* effect of bacteriocin-incorporated agar edible coatings applied on cheese. Int Dairy J 97:92–98. 10.1016/j.idairyj.2019.05.016

[9] Nes IF, Diep DB, Håvarstein LS, Brurberg MB, Eijsink V, Holo H (1996) Biosynthesis of bacteriocins in lactic acid bacteria. Antonie van Leeuwenhoek 70(2–4):113–128. 10.1007/bf00395929 8879403 10.1007/BF00395929

[10] Rossouw M, Cripwell RA, Vermeulen RR, Van Staden AD, Van Zyl WH, Dicks LMT, Vijloen-Bloom M (2023) Heterologous expression of plantaricin 423 and mundticin ST4SA in *Saccharomyces cerevisiae*. Probiotics Antimicrob Proteins 16:845–861. 10.1007/s12602-023-10082-6 37171691 10.1007/s12602-023-10082-6 PMC11126478

[11] Zhang T, Zhang Y, Li L, Jiang X, Chen Z, Zhao F, Yi Y (2022) Biosynthesis and production of class II bacteriocins of food-associated lactic acid bacteria. Fermentation 8(5):217. 10.3390/fermentation8050217

[12] Celik E, Calık P (2012) Production of recombinant proteins by yeast cells. Biotechnol Adv 30(5):1108–18. 10.1016/j.biotechadv.2011.09.011 21964262 10.1016/j.biotechadv.2011.09.011

[13] Eijsink VG, Skeie M, Middelhoven PH, Brurberg MB, Nes IF (1998) Comparative studies of class IIa bacteriocins of lactic acid bacteria. Appl Environ Microbiol 64(9):3275–3281. 10.1128/AEM.64.9.3275-3281.1998 9726871 10.1128/aem.64.9.3275-3281.1998 PMC106721

[14] Oppegård C, Fimland G, Anonsen JH, Nissen-Meyer J (2015) The pediocin PA-1 accessory protein ensures correct disulfide bond formation in the antimicrobial peptide pediocin PA-1. Biochemistry 54(19):2967–2974. 10.1021/acs.biochem.5b00164 25961806 10.1021/acs.biochem.5b00164

[15] Martínez JM, Kok J, Sanders JW, Hernández PE (2000) Heterologous coproduction of enterocin A and pediocin PA-1 by *Lactococcus lactis*: detection by specific peptide-directed antibodies. Appl Environ Microbiol 66(8):3543–9. 10.1128/AEM.66.8.3543-3549.2000 10919819 10.1128/aem.66.8.3543-3549.2000 PMC92183

[16] Klocke M, Mundt K, Idler F, Jung S, Backhausen JE (2005) Heterologous expression of enterocin A, a bacteriocin from *Enterococcus faecium*, fused to a cellulose-binding domain in *Escherichia coli* results in a functional protein with inhibitory activity against *Listeria*. Appl Microbiol Biotechnol 67(4):532–8. 10.1007/s00253-004-1838-5 15660219 10.1007/s00253-004-1838-5

[17] Borrero J, Kunze G, Jiménez JJ, Böer E, Gútiez L, Herranz C, Cintas LM, Hernández PE (2012) Cloning, production, and functional expression of the bacteriocin enterocin A, produced by *Enterococcus faecium* T136, by the yeasts *Pichia pastoris*, *Kluyveromyces lactis*, *Hansenula polymorpha*, and *Arxula adeninivorans*. Appl Environ Microbiol 78(16):5956–61. 10.1128/AEM.00530-12 22685156 10.1128/AEM.00530-12 PMC3406115

[18] Vermeulen RR, Van Staden AD, Dicks LMT (2020) Heterologous expression of the class IIa bacteriocins, plantaricin 423 and mundticin ST4SA, in *Escherichia coli* using green fluorescent protein as a fusion partner. Front Microbiol 11:1634. 10.3389/fmicb.2020.01634 32765464 10.3389/fmicb.2020.01634 PMC7381239

[19] Sambrook J, Fritsch EF, Maniatis T (1989) Molecular cloning: a laboratory manual. Cold Spring Harbor Laboratory Press, Cold Spring Harbor New York

[20] Cripwell RA, Rose S, Viljoen-Bloom M, van Zyl WH (2019) Improved raw starch amylase production by *Saccharomyces cerevisiae* using codon optimisation strategies. FEMS Yeast Res 19(2). 10.1093/femsyr/foy127 10.1093/femsyr/foy127 30535120

[21] Cho KM, Yoo YJ, Kang HS (1999) Δ-integration of endo/exoglucanase and β-glucosidase genes into the yeast chromosomes for direct conversion of cellulose to ethanol. Enzyme Microb Technol 25:23–30. 10.1016/S0141-0229(99)00011-3

[22] Schägger H (2006) Tricine–SDS-PAGE. Nat Protoc 1(1):16–22. 10.1038/nprot.2006.4 17406207 10.1038/nprot.2006.4

[23] Chevallet M, Luche S, Rabilloud T (2006) Silver staining of proteins in polyacrylamide gels. Nat Protoc 1(4):1852–8. 10.1038/nprot.2006.288 17487168 10.1038/nprot.2006.288 PMC1971133

[24] Baindara P, Chaudhry V, Mittal G, Liao LM, Matos CO, Khatri N, Franco OL, Patil PB, Korpole S (2016) Characterization of the antimicrobial peptide penisin, a class Ia novel lantibiotic from *Paenibacillus* sp. strain A3. Antimicrob Agents Chemother 60(1): 580–591. 10.1128/aac.01813-15 10.1128/AAC.01813-15 PMC4704198 26574006

[25] Clinical and Laboratory Standards Institute (2012) Methods for dilution antimicrobial susceptibility tests for bacteria that grow aerobically; approved standard. https://www.techstreet.com/mss/products/preview/1823012

[26] Pluskal T, Castillo S, Villar-Briones A, Oresic M (2010) MZmine 2: modular framework for processing visualizing, and analyzing mass spectrometry-based molecular profile data. BMC Bioinformatics 11:395. 10.1186/1471-2105-11-395 20650010 10.1186/1471-2105-11-395 PMC2918584

[27] Chen ZL, Meng JM, Cao Y, Yin JL, Fang RQ, Fan SB, Liu C, Zeng WF, Ding YH, Tan D, Wu L, Zhou WJ, Chi H, Sun RX, Dong MQ, He SM (2019) A high-speed search engine pLink 2 with systematic evaluation for proteome-scale identification of cross-linked peptides. Nat Commun 10:3404. 10.1038/s41467-019-11337-z 31363125 10.1038/s41467-019-11337-z PMC6667459

[28] Anane E, van Rensburg E, Görgens JF (2013) Optimisation and scale-up of α-glucuronidase production by recombinant *Saccharomyces cerevisiae* in aerobic fed-batch culture with constant growth rate. Biochem Eng J 81:1–7. 10.1016/j.bej.2013.09.012

[29] Vermeulen RR, Deane S, Dicks LMT, Rohwer J, van Staden AD (2021) Manganese privation-induced transcriptional upregulation of the class IIa bacteriocin plantaricin 423 in *Lactobacillus plantarum* strain 423. Appl Ind Microbiol 87(21):e00976-21. 10.1128/AEM.00976-21 10.1128/AEM.00976-21 PMC8516046 34406833

[30] LaMantia ML, Lennarz WJ (1993) The essential function of yeast protein disulfide isomerase does not reside in its isomerase activity. Cell 74(5):899–908. 10.1016/0092-8674(93)90469-7 8374956 10.1016/0092-8674(93)90469-7

[31] Pathak N, Salas-Auvert R, Ruche G, Janna MH, McCarthy D, Harrison RG (1995) Comparison of the effects of hydrophobicity amphiphilicity and alpha-helicity on the activities of antimicrobial peptides. Proteins 22(2):182–6. 10.1002/prot.340220210 7567965 10.1002/prot.340220210

[32] Grimsey E, Collis DWP, Mikut R (1862) Hilpert K (2020) The effect of lipidation and glycosylation on short cationic antimicrobial peptides. Biochim Biophys Acta - Biomembr 8:183195. 10.1016/j.bbamem.2020.183195 10.1016/j.bbamem.2020.183195 32130974

[33] Makovitzki A, Avrahami D, Shai Y (2006) Ultrashort antibacterial and antifungal lipopeptides. Proc Natl Acad Sci USA 103(43):15997–16002. 10.1073/pnas.0606129103 17038500 10.1073/pnas.0606129103 PMC1635116

[34] Shi SM, Di L (2022) Strategies to optimize peptide stability and prolong half-life. In: Jois, S.D. (eds) Peptide Therapeutics. AAPS Advances in the Pharmaceutical Sciences Series, vol 47. Springer, Cham. 10.1007/978-3-031-04544-8_4

[35] Torosantucci R, Schöneich C, Jiskoot W (2014) Oxidation of therapeutic proteins and peptides: structural and biological consequences. Pharm Res 31(3):541–53. 10.1007/s11095-013-1199-9 24065593 10.1007/s11095-013-1199-9

[36] Hansen IKØ, Isaksson J, Poth AG, Hansen KØ, Andersen AJC, Richard CSM, Blencke HM, Stensvåg K, Craik DJ, Haug T (2020) Isolation and characterization of antimicrobial peptides with unusual disulfide connectivity from the colonial ascidian *Synoicum turgens*. Mar Drugs 18(1):51. 10.3390/md18010051 31940927 10.3390/md18010051 PMC7024374

[37] Johnsen L, Fimland G, Eijsink V, Nissen-Meyer J (2000) Engineering increased stability in the antimicrobial peptide pediocin PA-1. Appl Environ Microbiol 66(11):4798–802. 10.1128/AEM.66.11.4798-4802.2000 11055926 10.1128/aem.66.11.4798-4802.2000 PMC92382

[38] Goumon Y, Strub JM, Moniatte M, Nullans G, Poteur L, Hubert P, Van Dorsselaer A, Aunis D, Metz-Boutigue MH (1996) The C-terminal bisphosphorylated proenkephalin-A-(209–237)-peptide from adrenal medullary chromaffin granules possesses antibacterial activity. Eur J Biochem 235(3):516–25. 10.1111/j.1432-1033.1996.t01-1-00516.x 8654396 10.1111/j.1432-1033.1996.t01-1-00516.x

[39] De Deken RH (1966) The Crabtree effect: a regulatory system in yeast. Microbiology 44:149–156. 10.1099/00221287-44-2-149 10.1099/00221287-44-2-149 5969497

