# Supplementary material for: Expression of Enterocin A in Saccharomyces cerevisiae
Source: Probiotics Antimicrob Proteins. 2025 Aug 30;18(3):3991–4011. doi: 10.1007/s12602-025-10686-0 (PMC13176010; doi:10.1007/s12602-025-10686-0)
Supplement: Supplementary file 1 — Supplementary file1 (DOCX 15780 KB) [file 12602_2025_10686_MOESM1_ESM.docx]

**Supplementary Information**

**Engineering *Saccharomyces cerevisiae* to produce enterocin A**

Michelle Rossouw^a^, Gerhardt Coetzee^b^, Rosemary A. Cripwell^a^, Willem H. van Zyl^a^, Leon M.T. Dicks^a^, Carla L. Ritter^a^ and Marinda Viljoen-Bloom^a^*

^a^Department of Microbiology, Stellenbosch University, Private Bag X1, Matieland, 7602, South Africa.

^b^Department of Chemical Engineering, Stellenbosch University, Private Bag X1, Matieland, Stellenbosch, 7602, South Africa.

*Corresponding author: Marinda Viljoen-Bloom; Phone: +27218085859; E-mail: mv4@sun.ac.za

**Fig. S1** Summary of the native and codon-optimised nucleotide and amino acid sequences of enterocin A. The changes between the native and codon-optimised nucleotide sequences of the bacteriocins are indicated in red.

**Table S1**: PCR primers used in this study

| **Primer name** | **Sequence (5’ – 3’)** |
| --- | --- |
| **PCR** |  |
| pMR-EntA_Opt-F | GTATCTTTGGATAAAAGAGAGGCTGAAGCTACTACACATTCTGGTAAATATTACG |
| pMR-EntA_Opt-R | GGACTAGAAGGCTTAATCAAAAGCTCTCGAGTTAACATTTACCAGGAATAGCACCA |


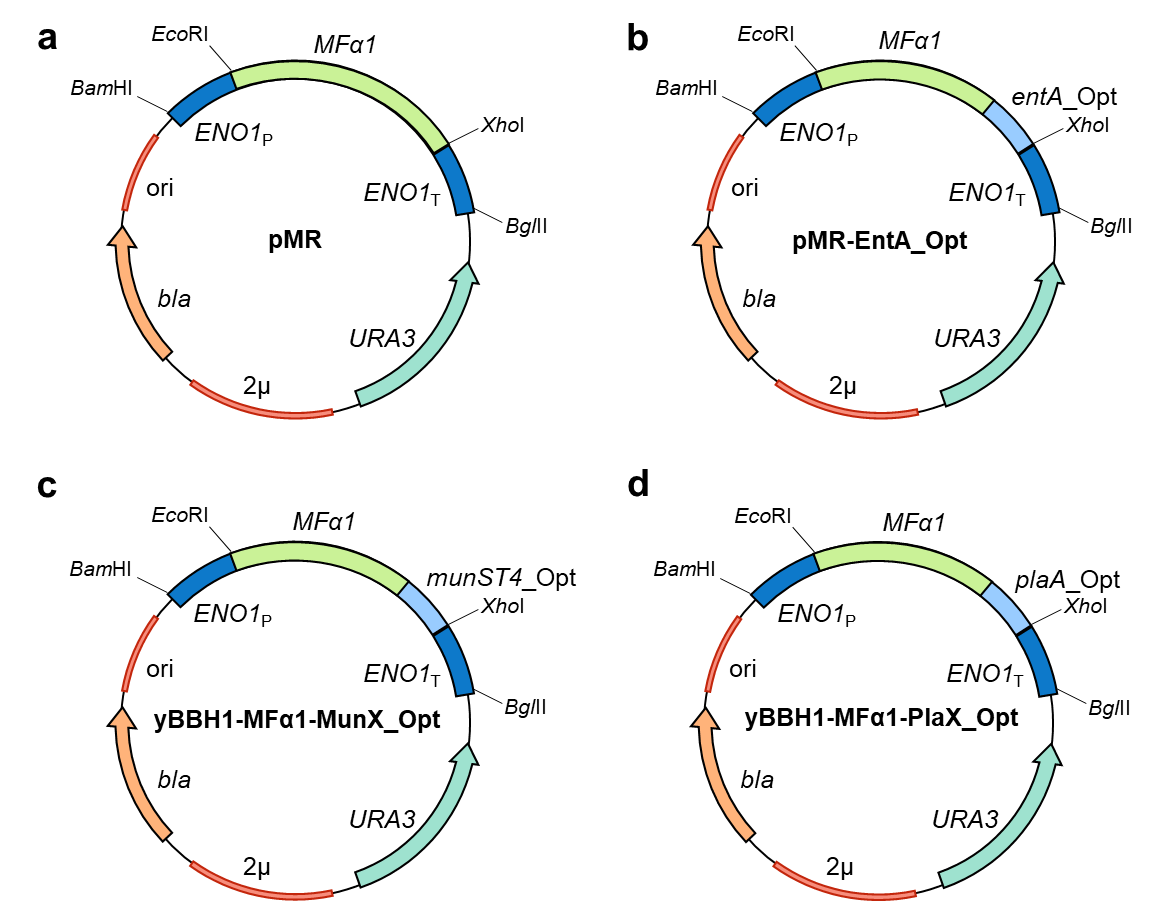


**Fig. S2** Schematic representation of (**a**) pMR with the MFα1 secretion signal served as the backbone for (**b**) cloning of the codon-optimised *entA* gene. The previously constructed plasmids (**c**) yBBH1-MFα1-MunX_Opt and (**d**) yBBH1-MFα1-PlaX_Opt (Rossouw et al., 2023) were included for comparative purposes.

**Fig. S3** Operon structures of class IIa bacteriocins. (**a**) Operon structure of native bacteriocins containing one or (**b**) two disulphide bonds. Light blue arrows indicate precursor peptides genes; light green, immunity genes; black, accessory disulphide bond modification proteins; red, transporter-related genes; orange, response regulators; purple, histidine kinases; pink, transcriptional regulators; dark green, transposases; dark blue, DNA mobility/transfer proteins; grey, induction factors; and yellow, genes with diverse functions.

*
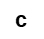
*

| **Peptide** | **GC content (%)** | | **Codon Bias Index (CBI)** | | **Codon Adaptation Index (CAI)** | |
| --- | --- | --- | --- | --- | --- | --- |
|  | Nat | Opt | Nat | Opt | Nat | Opt |
| Enterocin A | 40.10 | 40.17 | -0.07 | 0.48 | 0.78 | 0.92 |

**Fig. S4** The distribution of the relative codon usage frequency along the length of the (**a**) *entA* and (**b**) *entA_Opt* genes expressed in *S. cerevisiae*. (**c**) Summary of the codon usage bias of enterocin A in *S. cerevisiae.*

**
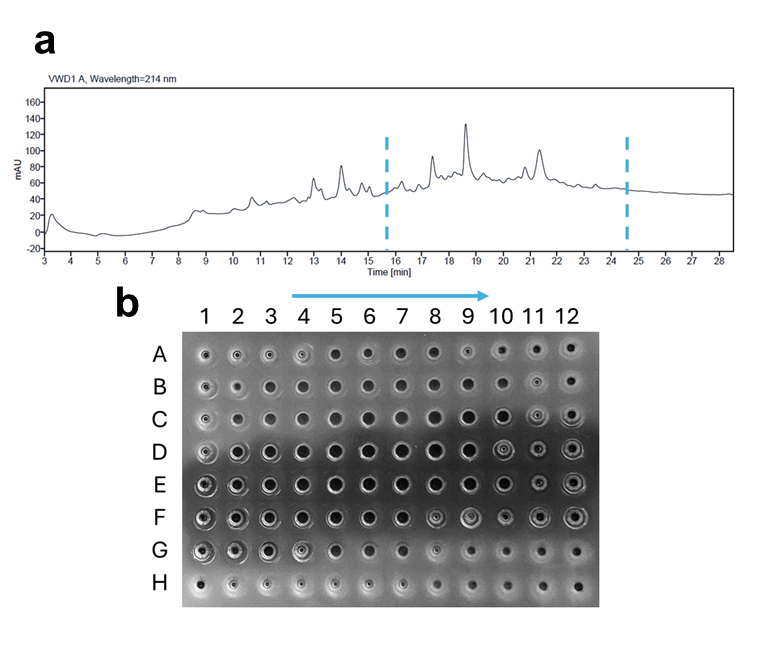
**

**Fig. S5** HPLC purification (C8 column) of EntA_Opt produced by recombinant Y294. **(a)** HPLC chromatogram of EntA_Opt with fractions collected and **(b)** anti-listerial activity of the fractions. The dashed blue lines indicate the active fractions on the chromatogram from 15.77 – 24.52 min. The blue arrow indicates the direction in which the fractions were spotted. All active fractions were combined, freeze-dried and weighed to determine the peptide concentration.


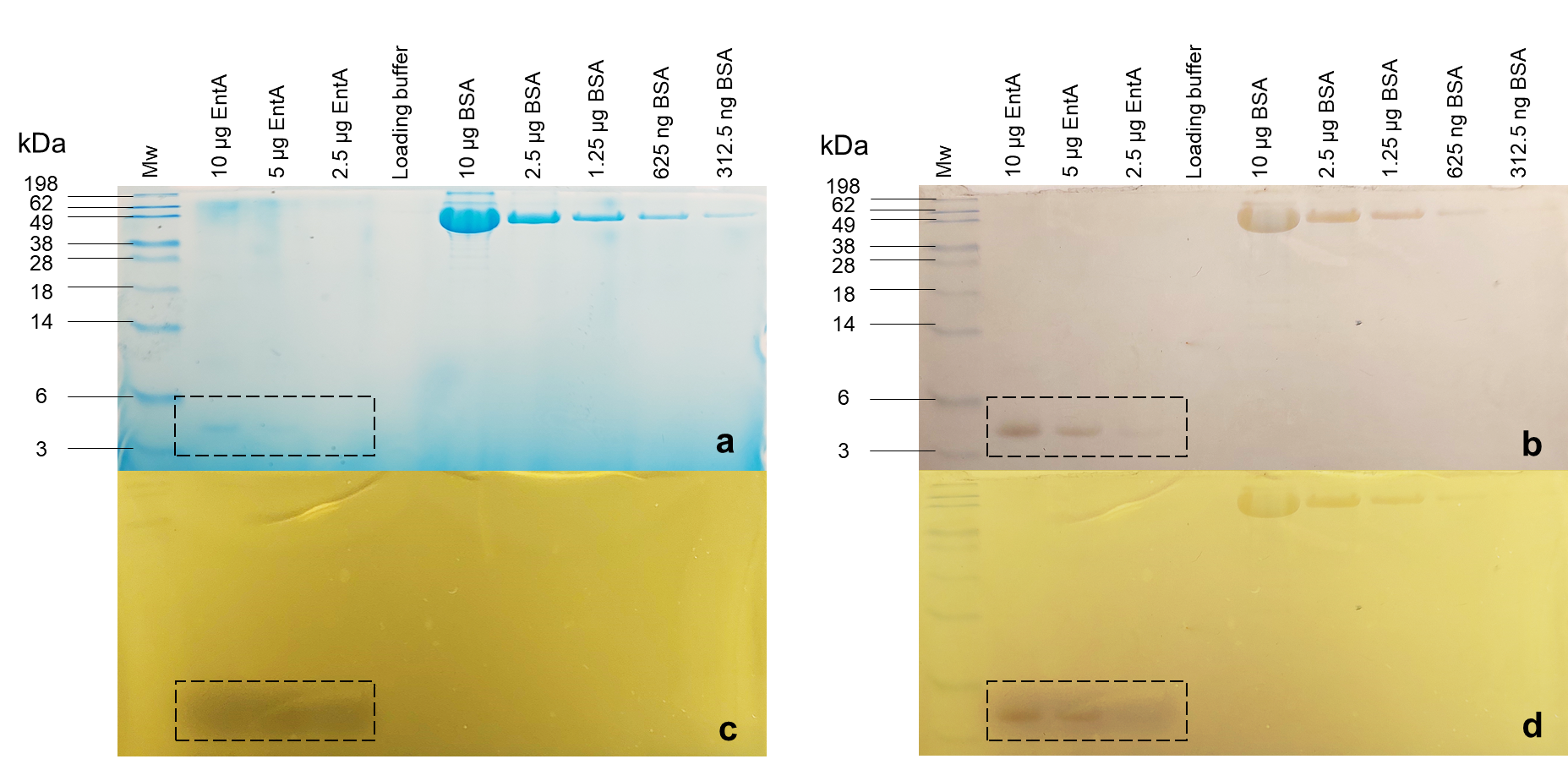


**Fig. S6** Tricine SDS-Page analysis and overlay of HPLC-purified enterocin A (EntA_Opt) from the Y294[MFα1-EntA_Opt] strain. Image **a** represents the Coomassie blue stained gel and image **b** represents the same gel that was destained and then silver stained. Image **c** represents the antilisterial overlay with inhibition zones indicated with the dashed box. Image **d** represents the superimposed gels. To confirm purity, 10 µg – 2.5 µg of HPLC-purified EntA was loaded onto the gel. Faint bands were detected with Coomassie staining, but were more prominent after silver staining. Antilisterial activity was detected for EntA. A serial dilution of bovine serum albumin (BSA) of the same concentrations as the peptide was loaded onto the gel as a control. No larger bands were detected from the purified peptide samples, indicating at least 95% purity.


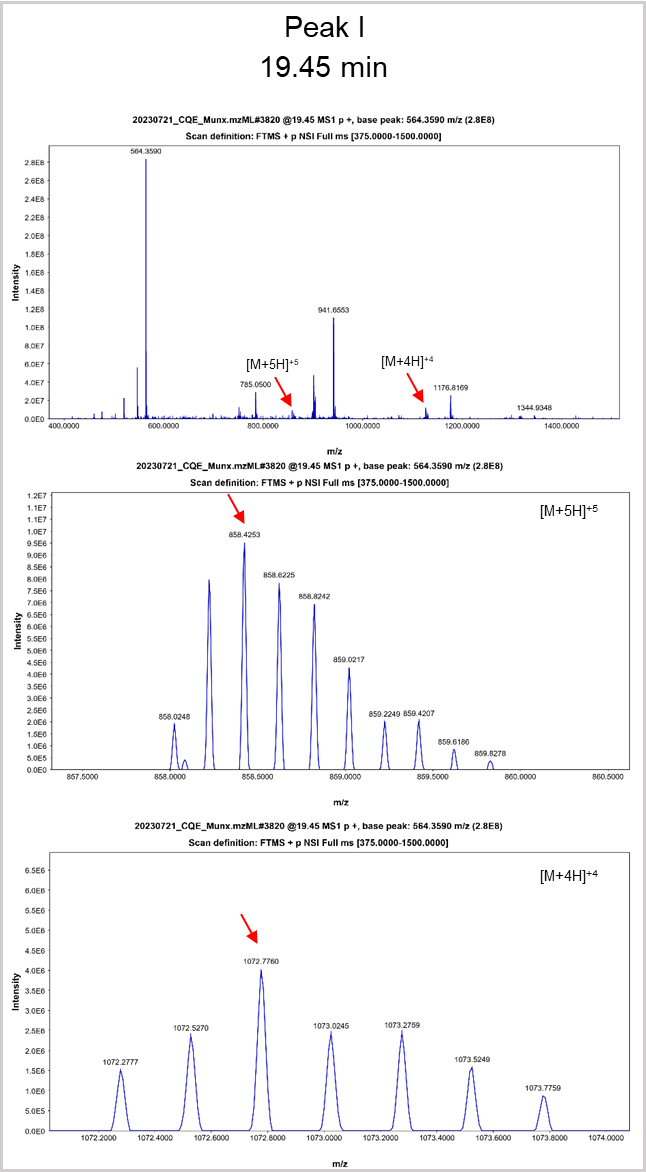


**a**

**b**

**c**

**Fig. S7** Accurate mass determination of MunX_Opt at Peak I (retention time 19.45). (**a**) Mass spectra indicating the monoisotopic ions (red arrows) observed for MunX_Opt. Monoisotopic ions observed for MunX_Opt carrying (**b**) +5 charges [M+5H]^+5^
(*m/z* 858.4253) and (**c**) +4 charges [M+4H]^+4^ (*m/z* 1072.7760) corresponding to peptide species containing no disulfide bonds.

**
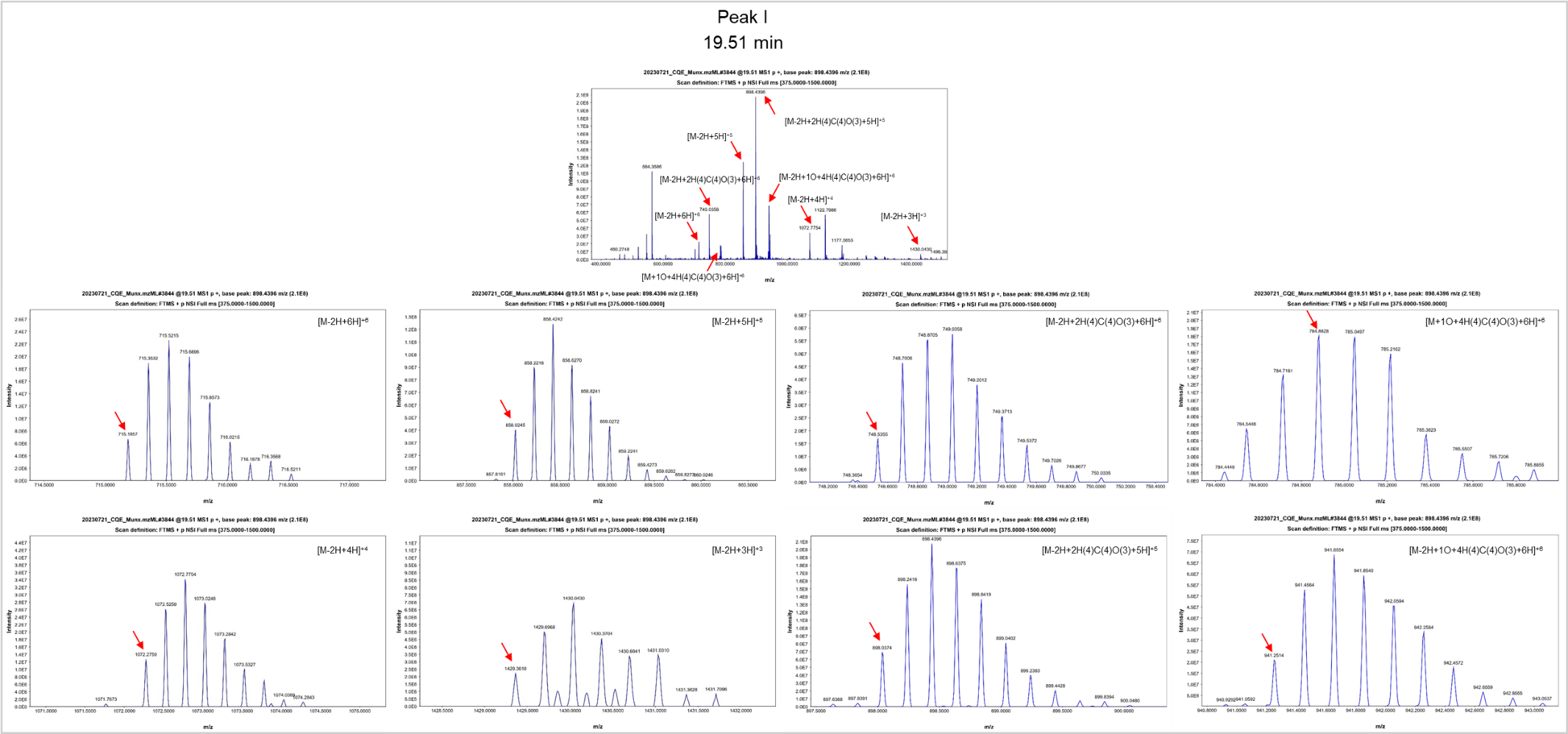
**

**a**

**e**

**d**

**c**

**b**

**g**

**f**

**h**

**f**

**Fig. S8** Accurate mass determination of MunX_Opt at Peak I (retention time 19.51). (**a**) Mass spectra indicating the monoisotopic ions (red arrows) observed for MunX_Opt. Monoisotopic ions observed for MunX_Opt corresponding to the species
(**b**) [M-2H+6H]^+6^ (*m/z* 715.1857), (**c**) [M-2H+5H]^+5^ (*m/z* 858.0245), (**d**) [M-2H+2H(4)C(4)O(3)+6H]^+6^ (*m/z* 748.5355),
(**e**) [M+1O+4H(4)C(4)O(3)+6H]^+6^ (*m/z* 784.8828), (**f**) [M-2H+4H]^+4^ (*m/z* 1072.2759), (**g**) [M-2H+3H]^+3^ (*m/z* 1429.3618),
(**h**) [M-2H+2H(4)C(4)O(3)+5H]^+5^ (*m/z* 898.0374), and (**i**) [M-2H+1O+4H(4)C(4)O(3)+6H]^+6^ (*m/z* 941.2514).


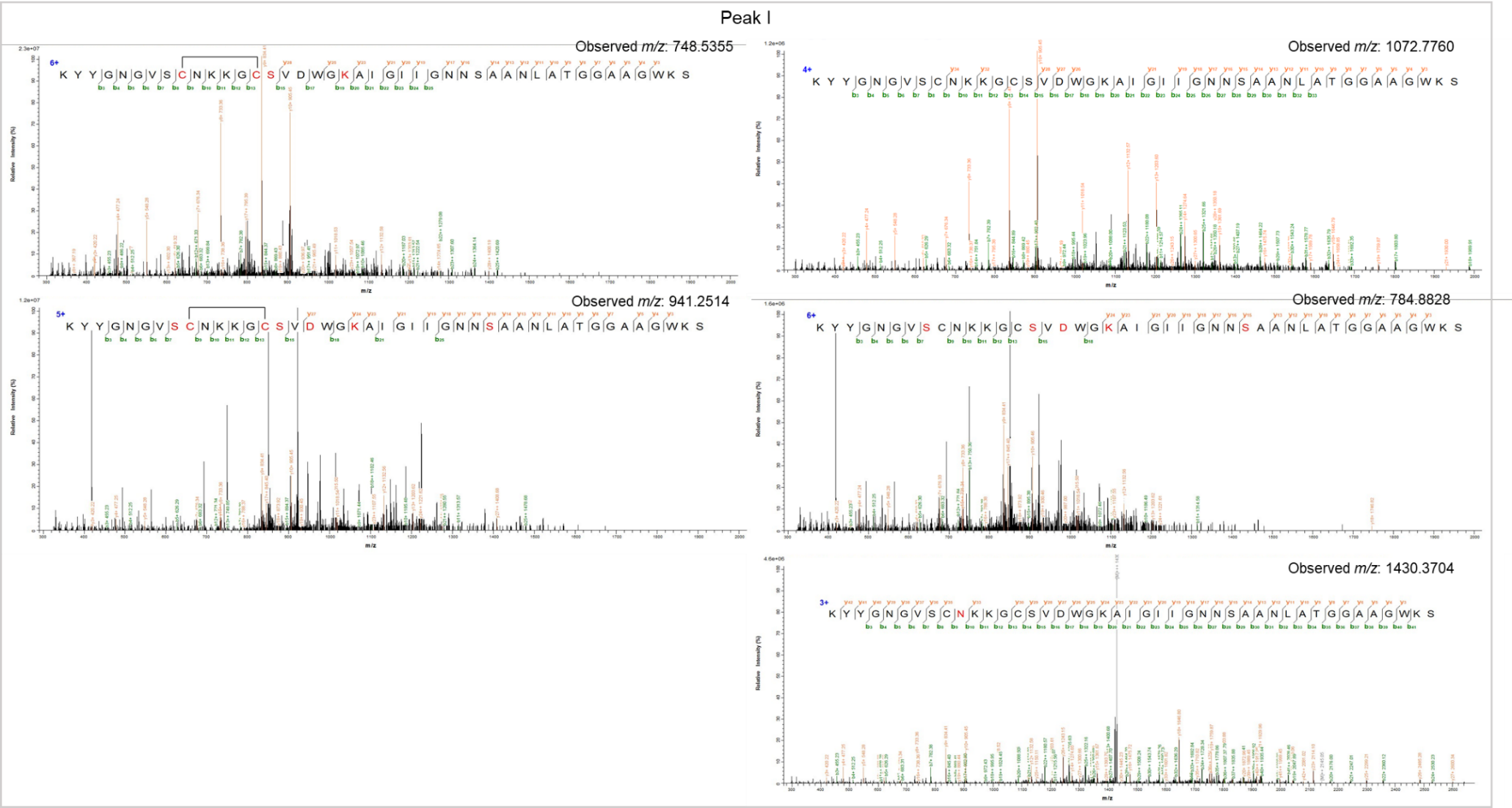


**Fig. S9** Tandem mass spectrometry of the monoisotopic parent ion from the mundticin ST4SA peptide envelopes observed at peak I (retention time 19.45 to 19.51 min). Collision-induced peptide fragmentation spectra confirmed the MunX_Opt peptide sequence at peak I with no disulphide bonds, with and without modifications. Adducts containing potential modifications such as deamination, succinylation, methylmalonylation and oxidation were observed and indicated in red at the putative modification site.


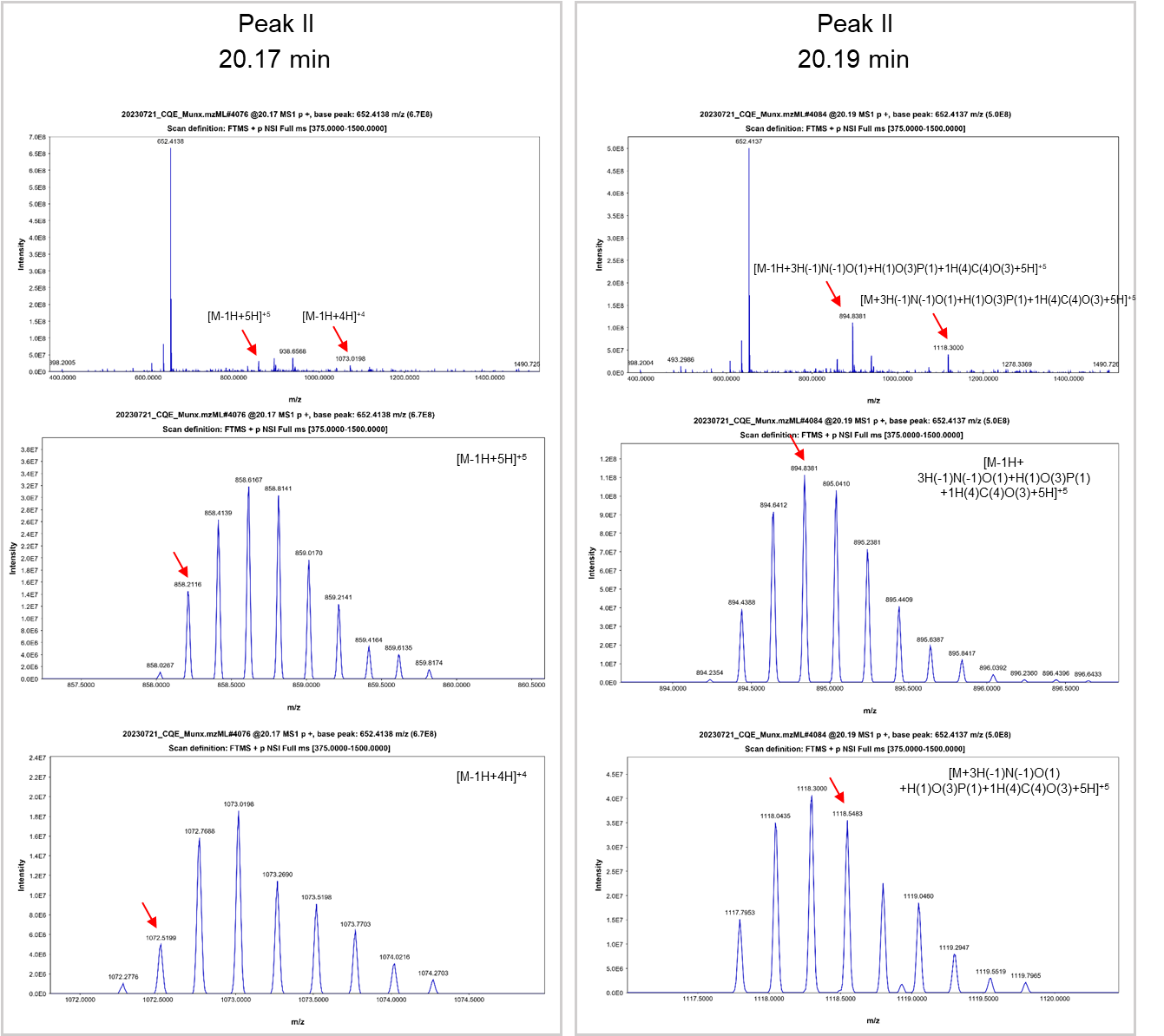


**e**

**f**

**d**

**c**

**b**

**a**

**Fig. S10** Accurate mass determination of MunX_Opt at Peak II (retention times 20.17 and 20.19). (**a**) Mass spectra at Rt 20.17 min and (**b**) mass spectra at Rt 20.19 min, indicating the monoisotopic ions (red arrows) observed for MunX_Opt. Monoisotopic ions observed for MunX_Opt corresponding to the species (**c**) [M-1H+5]^+5^ (*m/z* 858.2116), (**d**) [M-1H+4H]^+4^ (*m/z* 1072.5199), (**e**) [M-1H+3H(-1)N(-1)O(1)+H(1)O(3)P(1)+1H(4)C(4)O(3)+5H]+^5^ (*m/z* 894.8381), (**f**) [M+3H(-1)N(-1)O(1)+H(1)O(3)P(1)+1H(4)C(4)O(3)+5H]^+5^
(*m/z* 1118.5483).

**
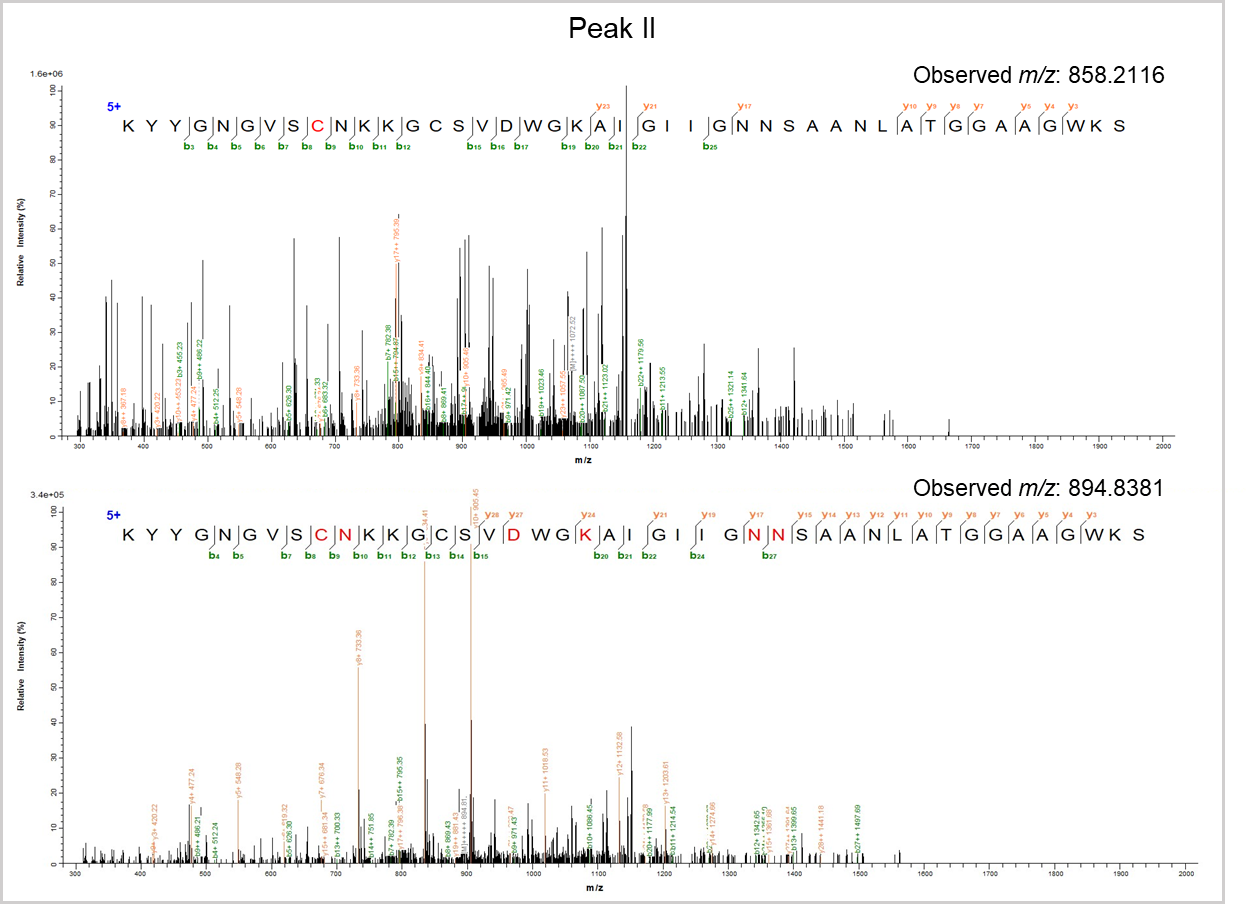
**

**Fig. S11** Tandem mass spectrometry of the monoisotopic parent ion from the mundticin ST4SA peptide envelopes observed at peak II (retention time 20.17 to 20.19 min). Collision-induced peptide fragmentation spectra confirmed the MunX_Opt peptide sequence at peak II with no disulphide bonds. Adducts containing potential modifications such as deamination, succinylation, methylmalonylation, oxidation and phosphorylation were observed and indicated in red at the putative modification site.


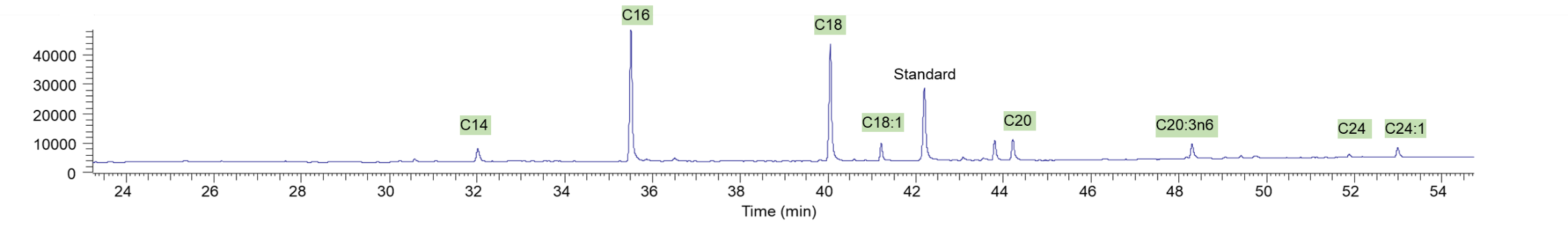


**a**

**b**

| **C number** | C12 | C14 | C16 | C16:1 | C18 | C18:1 (trans) | C18:1 (cis) | C18:2 (cis) | C20 | C20:1 | C18:3n3 | C22 | C20:3n6 | C22:2 | C20:5n3 | C24 | C24:1 |
| --- | --- | --- | --- | --- | --- | --- | --- | --- | --- | --- | --- | --- | --- | --- | --- | --- | --- |
| **Abundance (ppm)** | 0 | 0.273 | 18.658 | 0 | 16.095 | 0 | 0.017 | 0 | 2.972 | 0 | 0 | 0 | 4.289 | 0 | 0 | 0.214 | 0.141 |
| **Elution time** | - | - | 35.500 | - | 40.040 | 40.850 | 41.210 | - | 44.210 | - | - | - | 48.300 | - | - | 51.880 | 52.980 |
| **Fatty Acid** | Lauric Acid | Myristic acid | Palmitic acid | Palmitoleic acid | Stearic acid | Elaidic acid | Vaccenic acid | Linoleic acid | Arachidic acid | Eicosenoic acid | Linolenic acid | Behenic acid | Dihomo-γ-linolenic acid | Docosa-dienoic acid | Timno-donic acid | Ligno-ceric acid |  |

**Fig. S12** GC-MS analysis of the fatty acids bound to MunX_Opt. (**a**) GC-MS chromatogram of the fatty acids detected and (**b**) the abundance (in ppm) of the identified fatty acids.


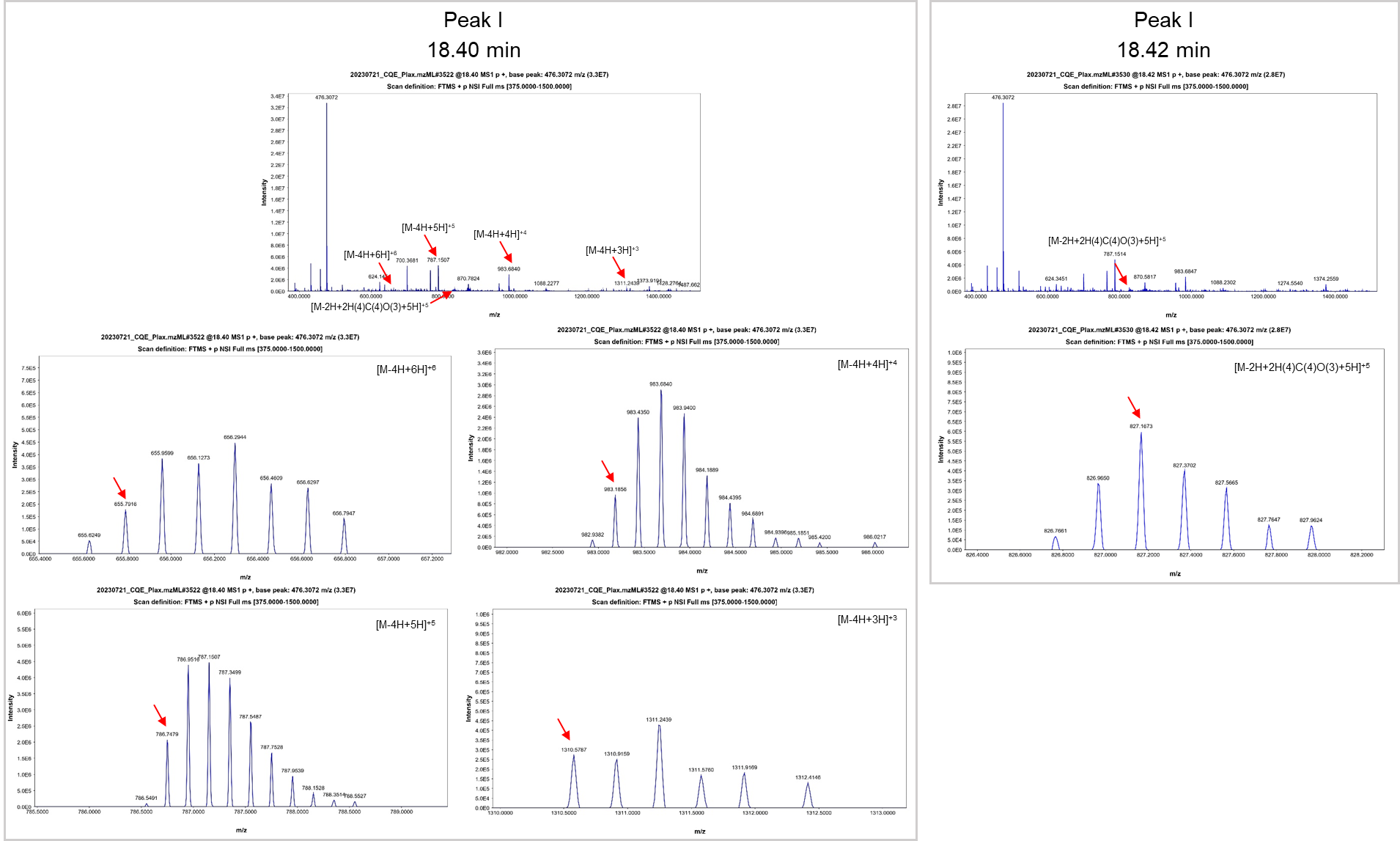


**Fig. S13** Accurate mass determination of PlaX_Opt at Peak I (retention time 18.40 – 18.42), with mass spectra indicating the monoisotopic ions (red arrows).

**
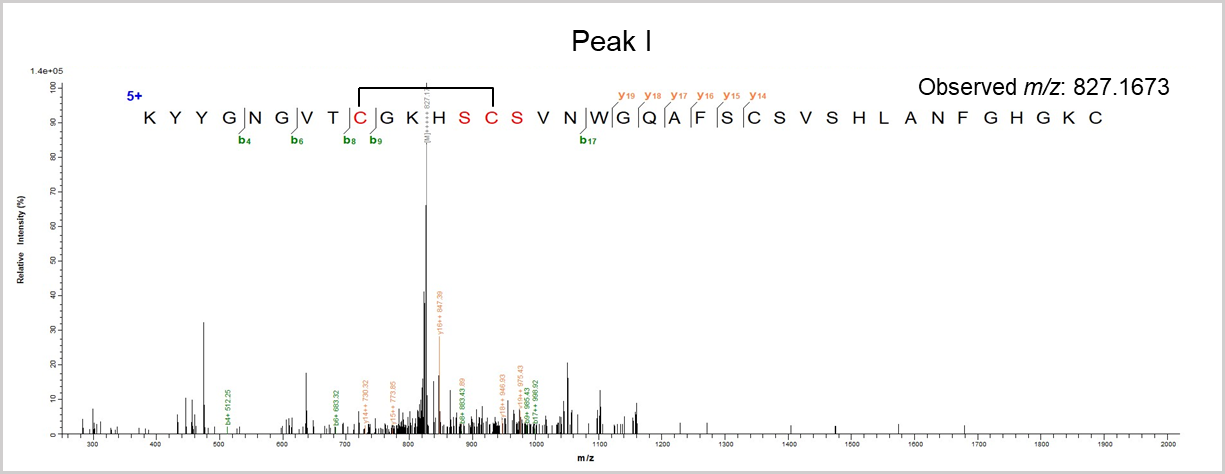
**

**Fig. S14** Tandem mass spectrometry of the monoisotopic parent ion from the PlaX_Opt envelopes observed at peak I (retention time 18.40 to 18.42 min) with Cys1 – Cys2 and Cys3 – Cys4 disulphide bonds. An adduct containing potential modifications, such as methylmalonylation, was observed and indicated in red at the putative modification sites.


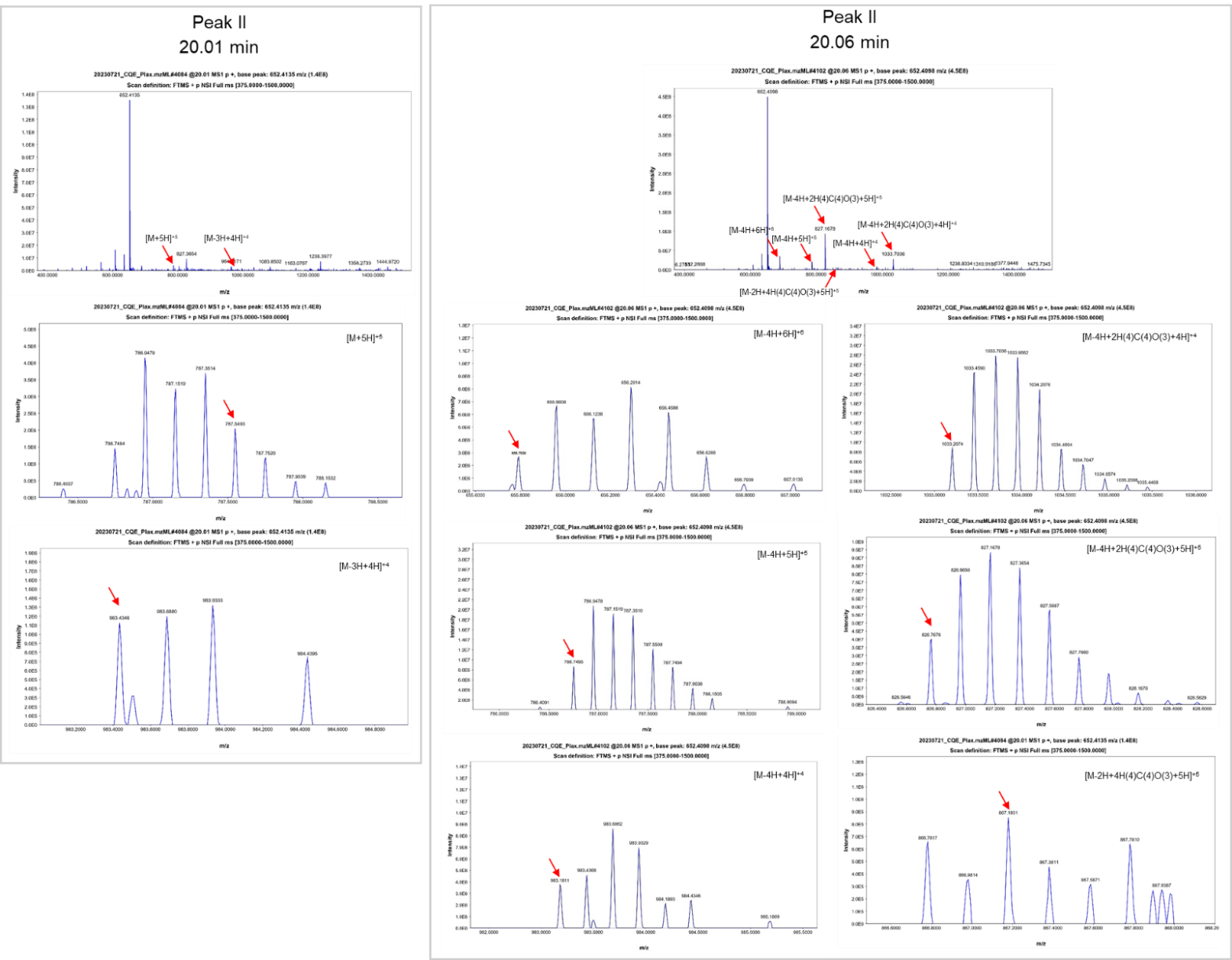


**Fig. S15** Accurate mass determination of PlaX_Opt at Peak II (retention time 20.01 – 20.06), with mass spectra indicating the monoisotopic ions (red arrows).

**
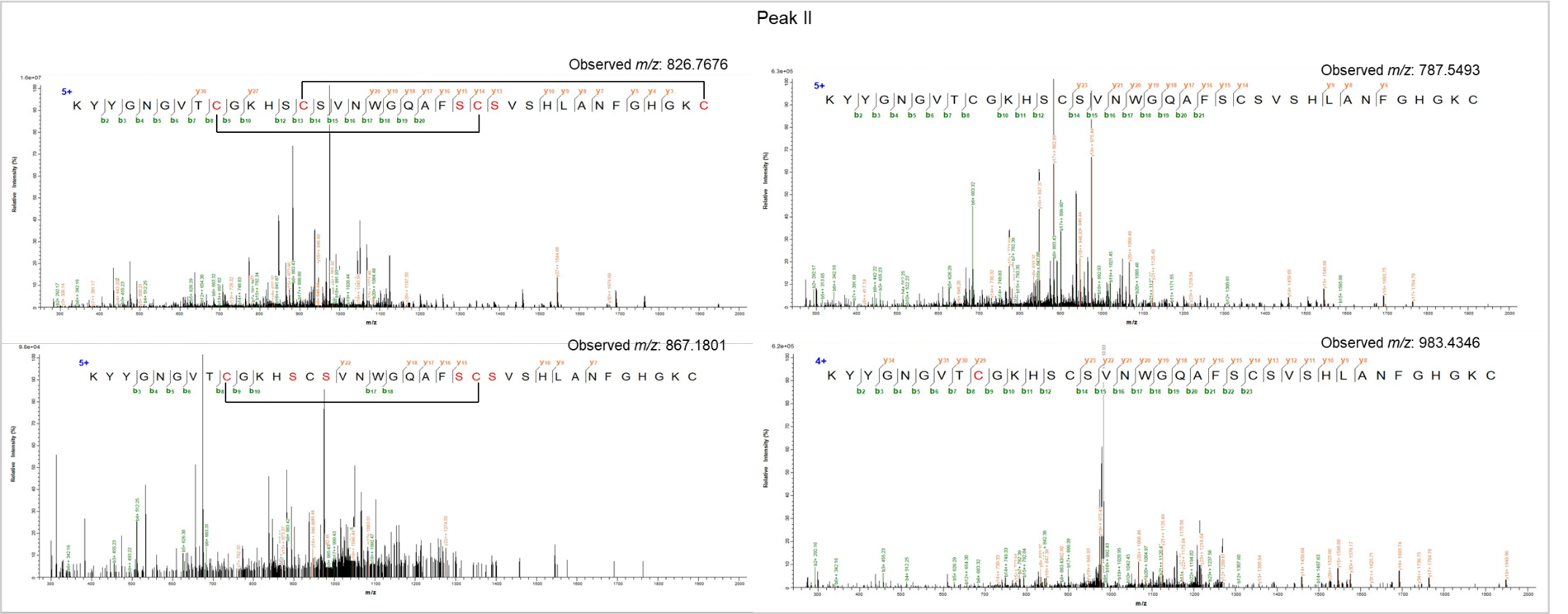
**

**Fig. S16** Tandem mass spectrometry of the monoisotopic parent ion from the PlaX_Opt envelopes observed at peak II (retention time 20.01 to 20.06 min) with Cys1 – Cys3 and Cys2 – Cys4 disulphide bonds. An adduct containing potential modifications, such as methylmalonylation, was observed and indicated in red at the putative modification sites.


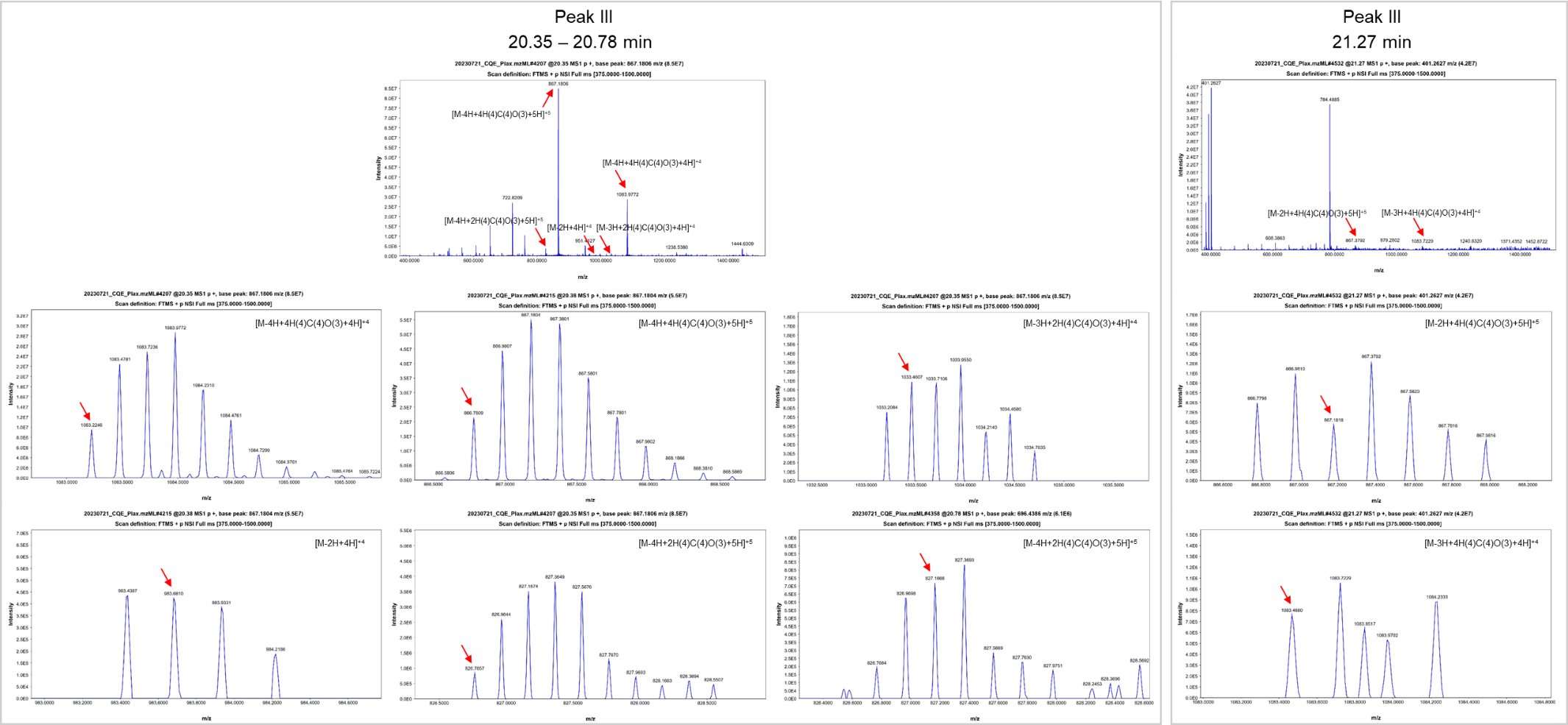


**Fig. S17** Accurate mass determination of PlaX_Opt at Peak III (retention time 20.35 – 21.27), with mass spectra indicating the monoisotopic ions (red arrows).

**
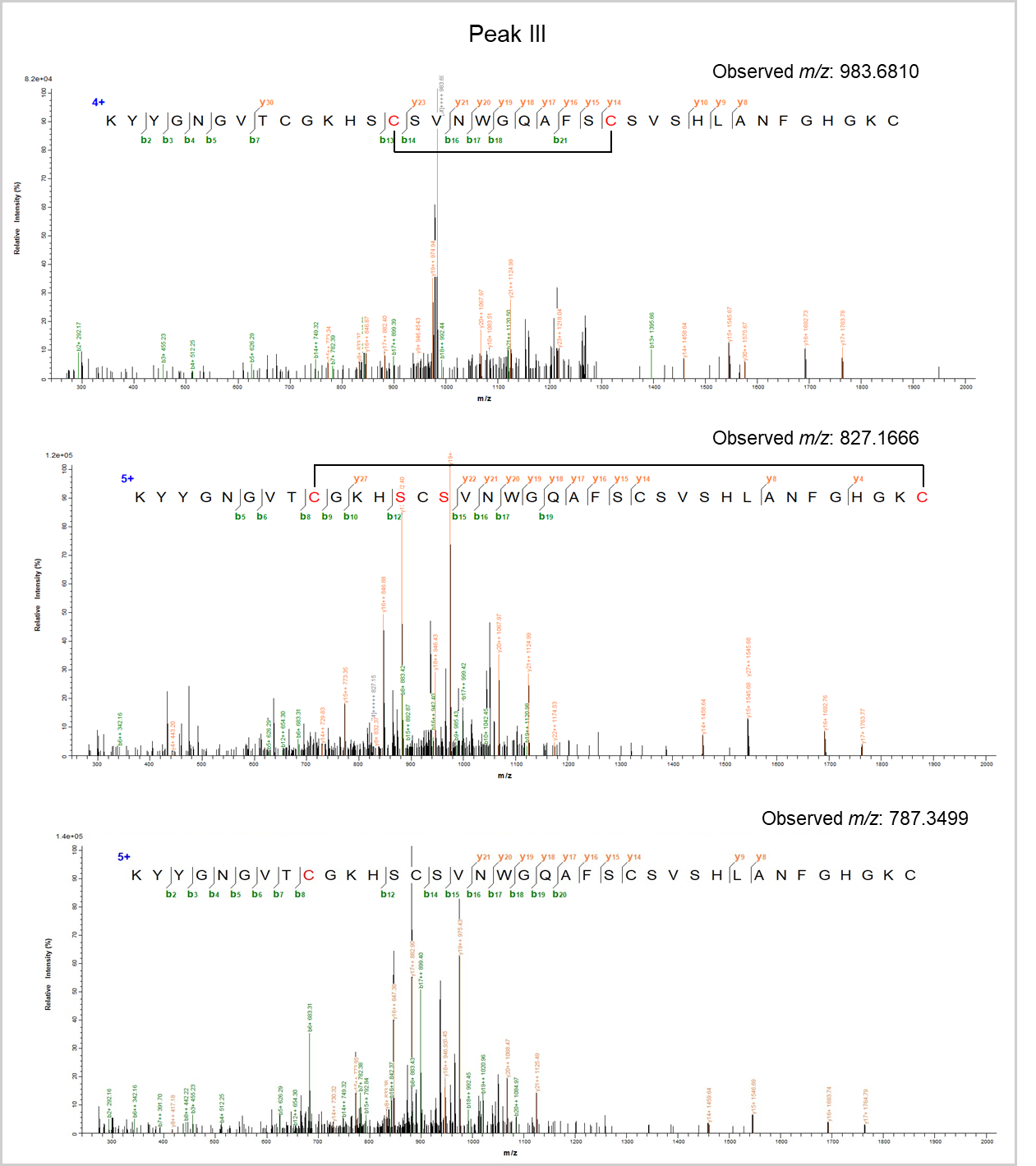
**

**Fig. S18** Tandem mass spectrometry of the monoisotopic parent ion from the PlaX_Opt envelopes observed at peak III (retention time 20.35 to 21.27 min) with Cys1 – Cys4 and Cys2 – Cys3 disulphide bonds. An adduct containing potential modifications, such as methylmalonylation, was observed and indicated in red at the putative modification sites.


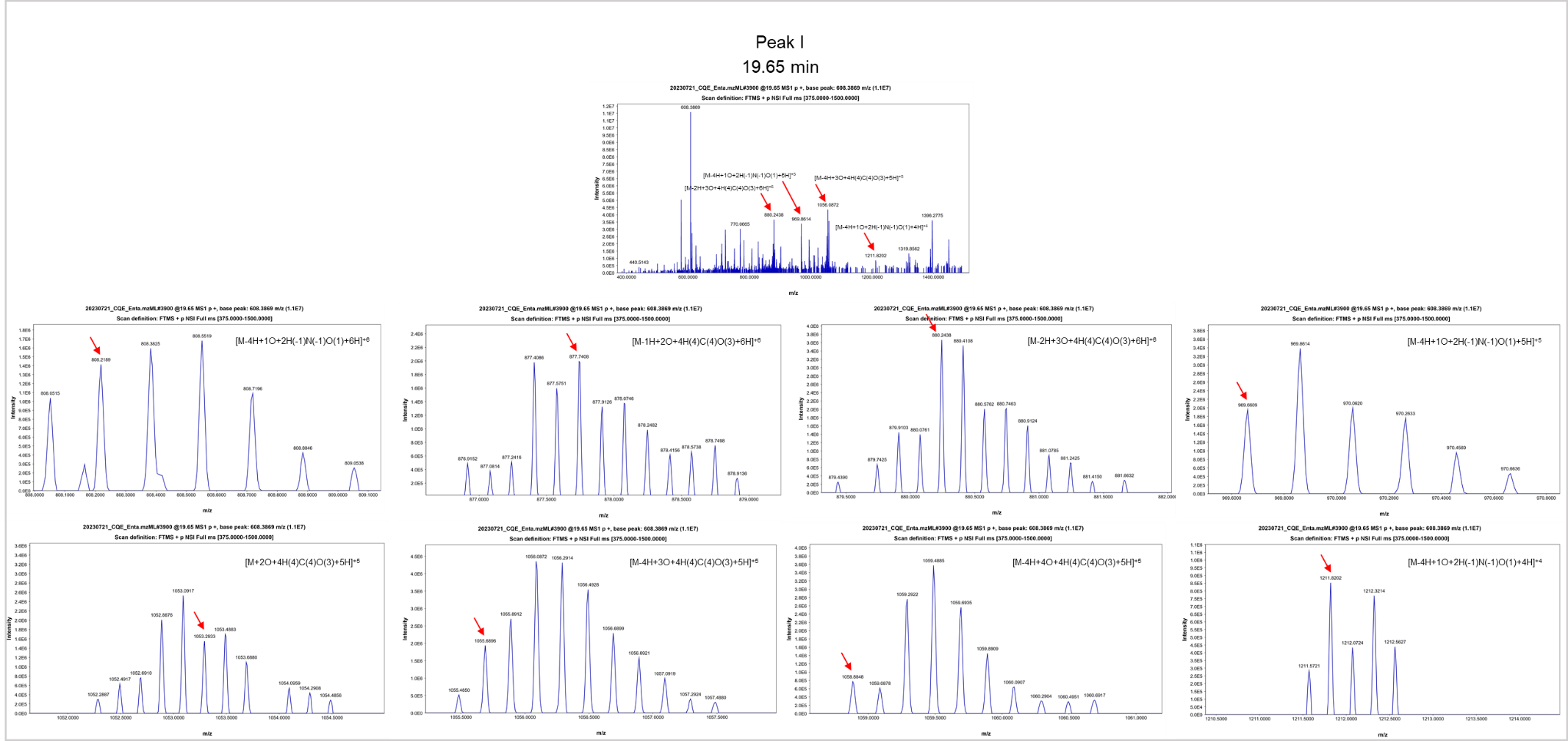


**a**

**e**

**d**

**c**

**b**

**h**

**i**

**g**

**f**

**Fig. S19** Accurate mass determination of EntA_Opt at Peak I (retention time 19.65). (**a**) Mass spectra indicating the monoisotopic ions (red arrows) observed for EntA_Opt. Monoisotopic ions observed for EntA_Opt corresponding to the species
(**b**) [M-4H+1O+2H(-1)N(-1)O(1)+6H]^+6^ (*m/z* 808.2189), (**c**) [M-1H+2O+4H(4)C(4)O(3)+6H]^+6^ (*m/z* 877.7408), (**d**) [M-2H+3O+4H(4)C(4)O(3)+6H]^+6^ (*m/z* 880.2438), (**e**) [M-4H+1O+2H(-1)N(-1)O(1)+5H]^+5^ (*m/z* 969.6609), (**f**) [M+2O+4H(4)C(4)O(3)+5H]^+5^ (*m/z* 1053.2933), (**g**) [M-4H+3O+4H(4)C(4)O(3)+5H]^+5^ (*m/z* 1055.6896), (**h**) [M-4H+4O+4H(4)C(4)O(3)+5H]^+5^ (*m/z* 1058.8848), and (**i**) [M-4H+1O+2H(-1)N(-1)O(1)+4H]^+4^ (*m/z* 1211.8202).


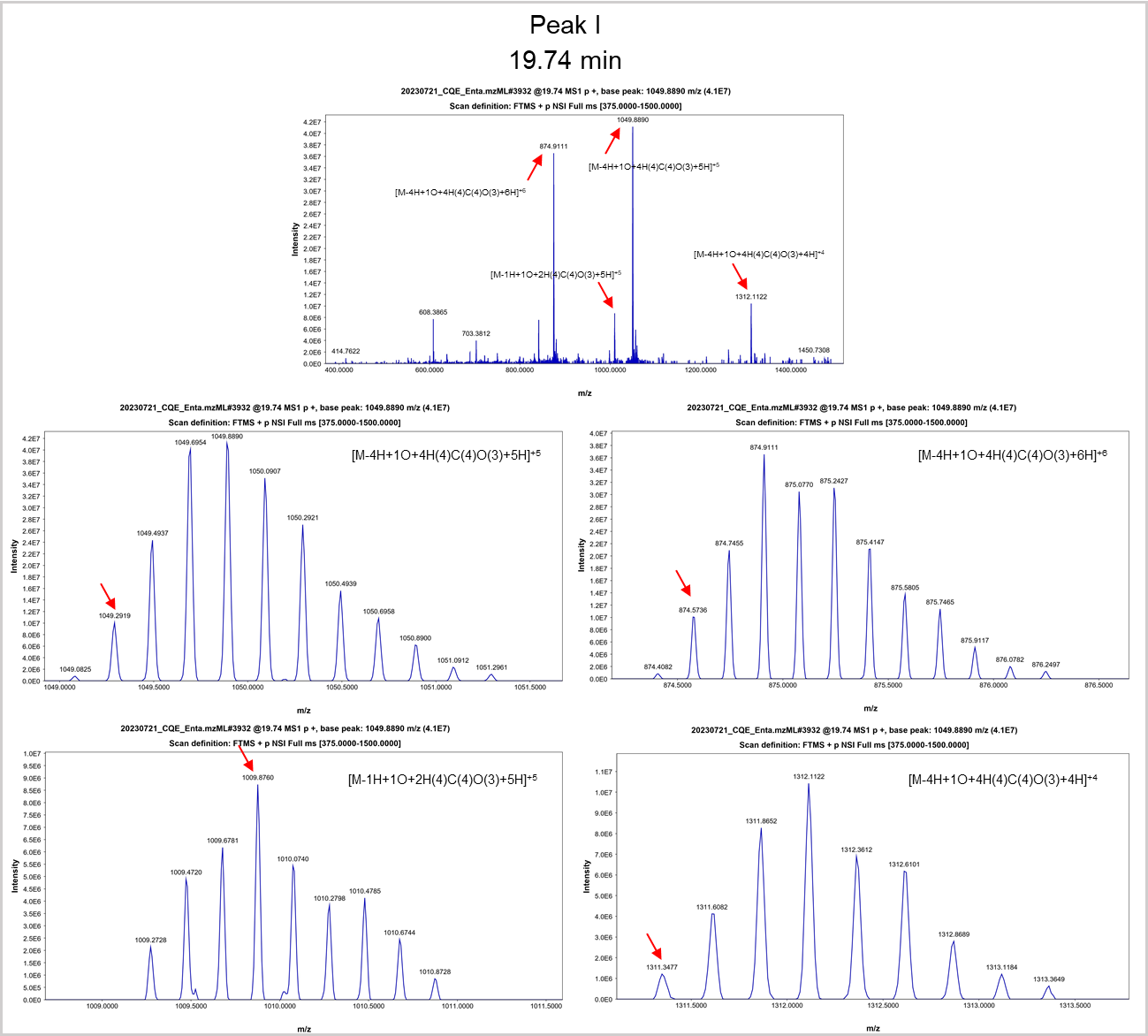


**e**

**d**

**c**

**b**

**a**

**Fig. S20** Accurate mass determination of EntA_Opt at Peak I (retention time 19.74). (**a**) Mass spectra indicating the monoisotopic ions (red arrows) observed for EntA_Opt. Monoisotopic ions observed for EntA_Opt corresponding to the species (**b**) [M-4H+1O+4H(4)C(4)O(3)+5H]^+5^ (*m/z* 1049.2919), (**c**) [M-4H+1O+4H(4)C(4)O(3)+6H]^+6^ (*m/z* 874.5736), (**d**) [M-1H+1O+2H(4)C(4)O(3)+5H]^+5^ (*m/z* 1009.8760), (**e**) [M-4H+1O+4H(4)C(4)O(3)+4H]^+4^ (*m/z* 1311.3477).

**
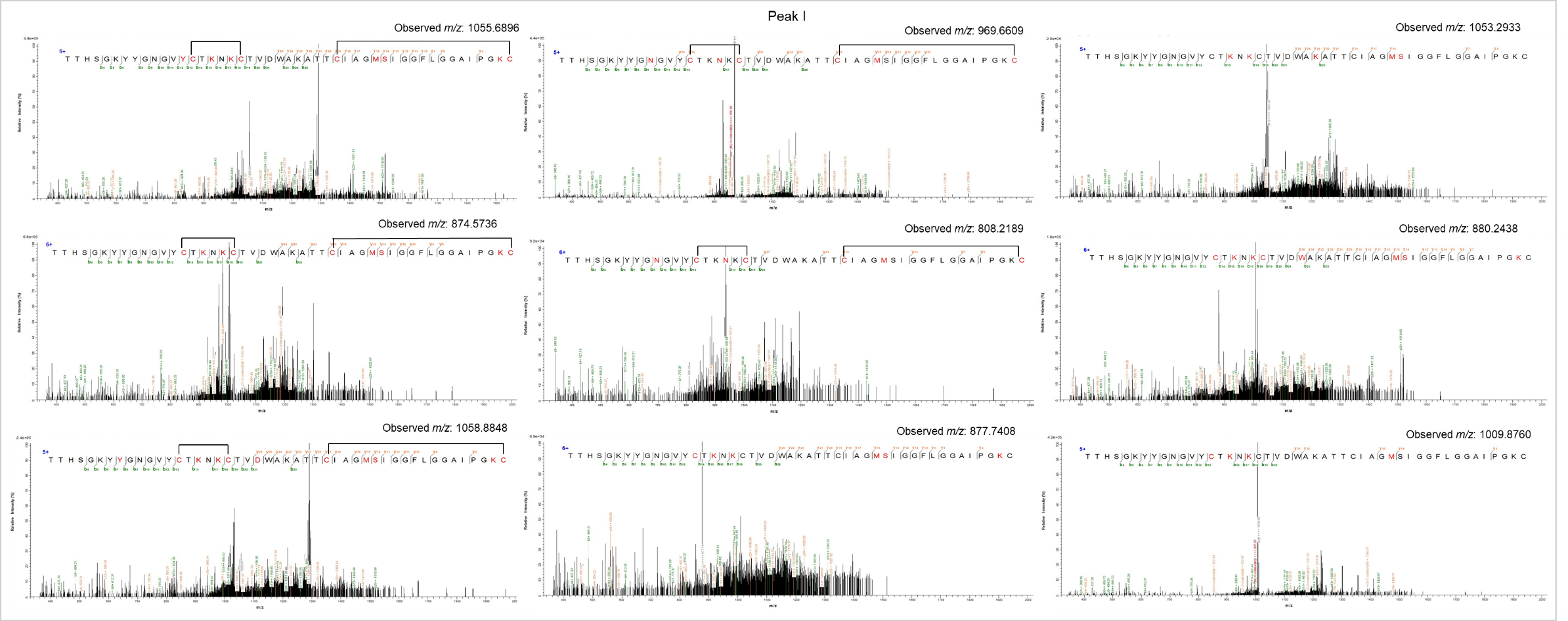
**

**Fig. S21** Tandem mass spectrometry of the monoisotopic parent ion from the EntA_Opt envelopes observed at peak I (retention time 19.65 to 19.74 min) with the formation of disulphide bonds between Cys1 – Cys2 and Cys3 – Cys4, and without disulphide bonds. Modifications are indicated in red.

**
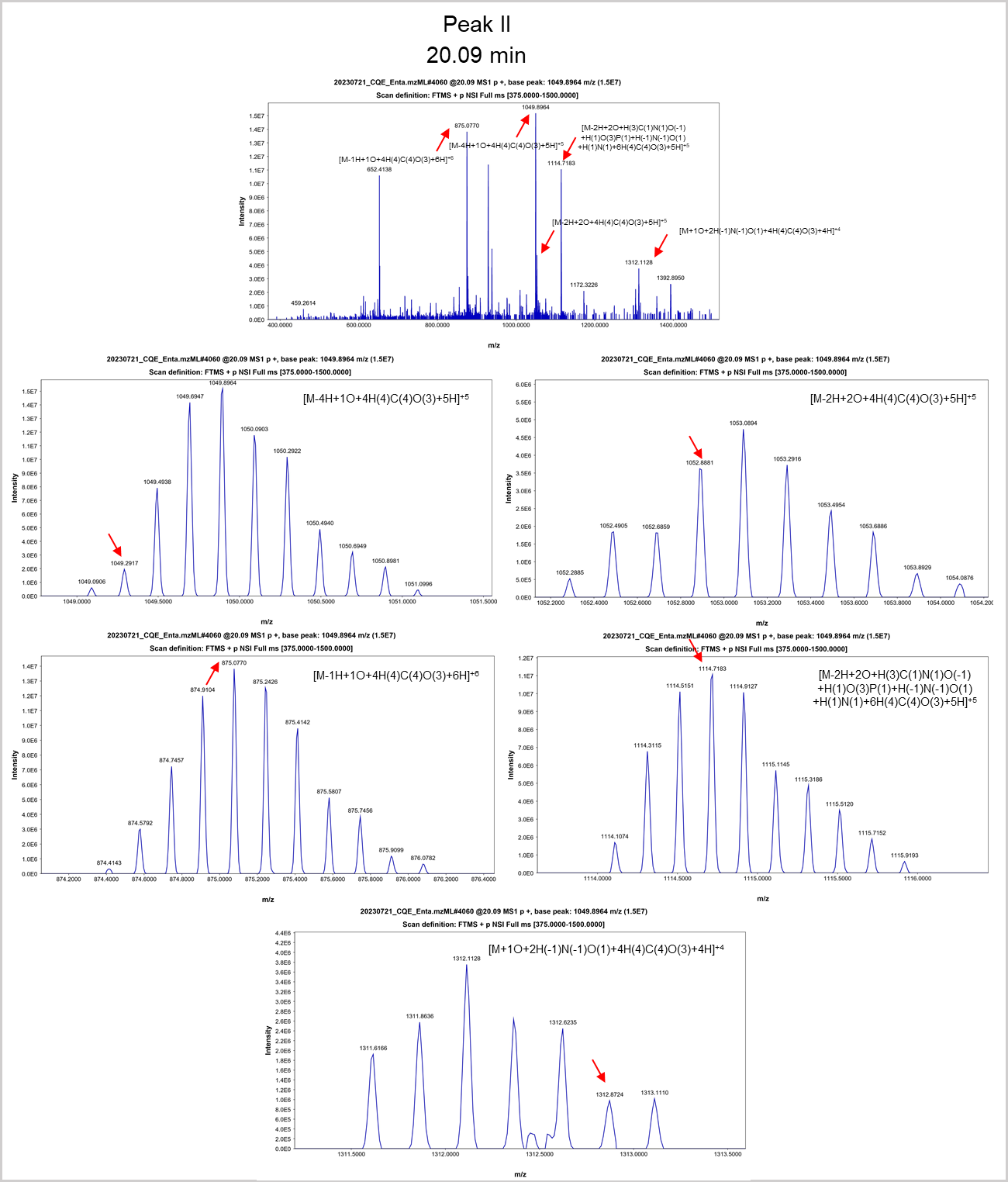
**

**f**

**e**

**d**

**c**

**b**

**a**

**Fig. S22** Accurate mass determination of EntA_Opt at Peak I (retention time 20.09). (**a**) Mass spectra indicating the monoisotopic ions (red arrows) observed for EntA_Opt. Monoisotopic ions observed for EntA_Opt corresponding to the species (**b**) [M-4H+1O+4H(4)C(4)O(3)+5H]^+5^ (*m/z* 1049.2917), (**c**) [M-2H+2O+4H(4)C(4)O(3)+5H]^+5^ (*m/z* 1052.8881), (**d**) [M-1H+1O+4H(4)C(4)O(3)+6H]^+6^ (*m/z* 875.0770), (**e**) [M-1H+2O+H(3)C(1)N(1)O(-1)+H(1)O(3)P(1)+H(1)N(1)+6H(4)C(4)O(3)+5H]^+5^ (*m/z* 1114.7184), (**f**) [M+1O+2H(-1)N(-1)O(1)+4H(4)C(4)O(3)+4H]^+4^ (*m/z* 1312.8724).

**f**

**e**

**d**

**
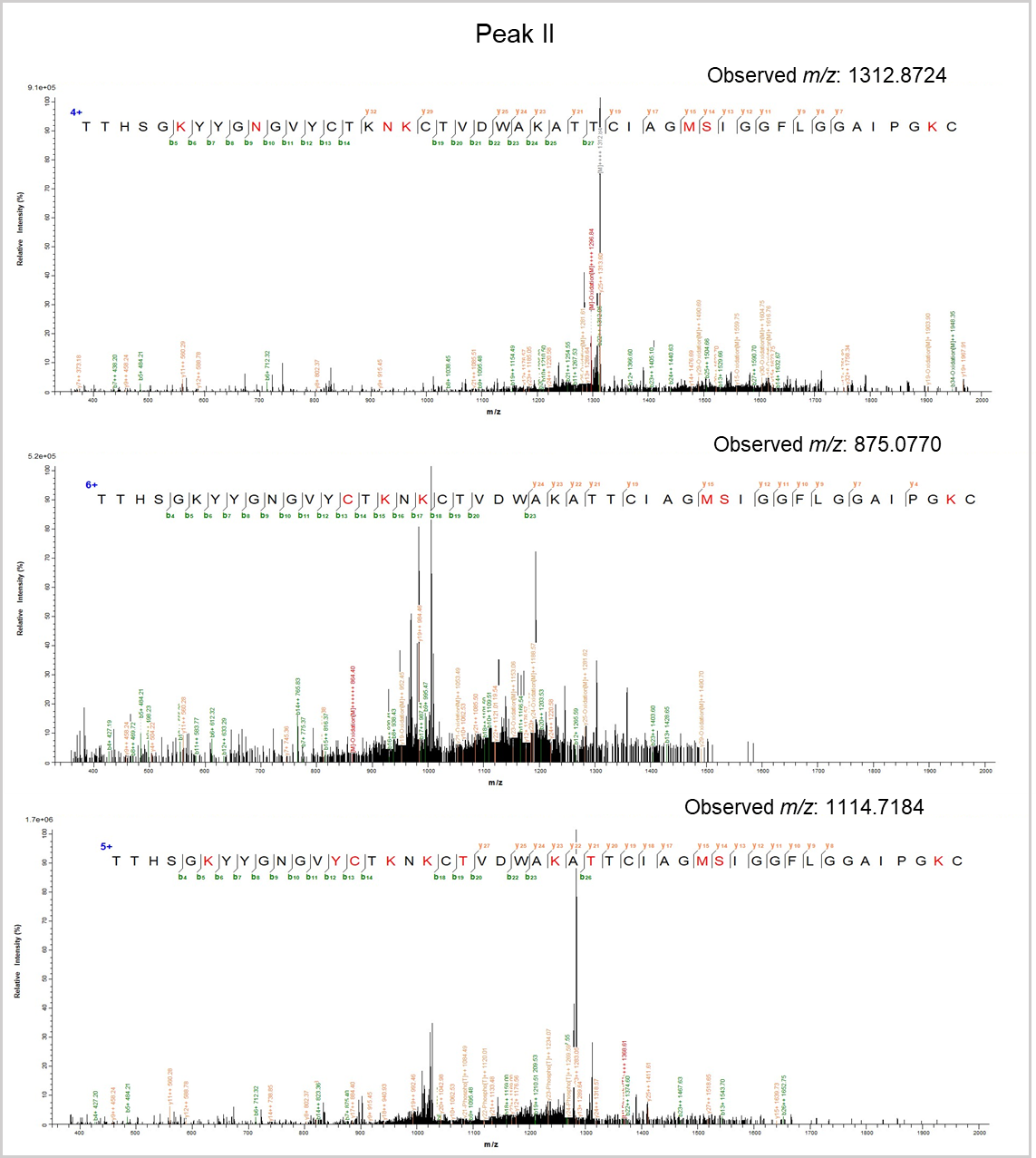
**

**Fig. S23** Tandem mass spectrometry of the monoisotopic parent ion from the peptide envelopes observed at peak II (retention time 20.09 min). Collision-induced peptide fragmentation spectra confirmed the EntA_Opt peptide sequence at peak II without disulphide bonds.

**
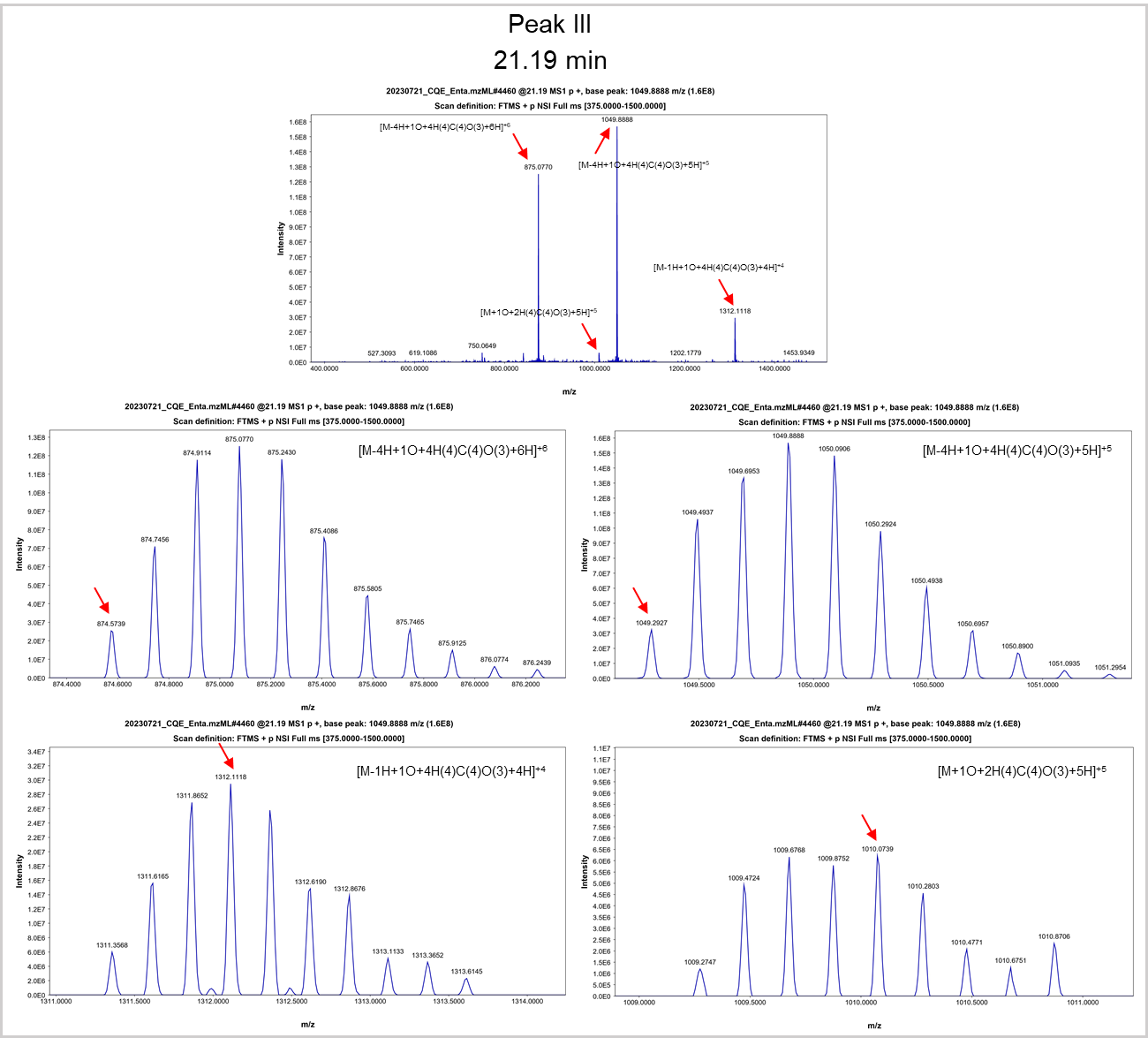
**

**e**

**d**

**c**

**b**

**a**

**Fig. S24** Accurate mass determination of EntA_Opt at Peak I (retention time 21.19). (**a**) Mass spectra indicating the monoisotopic ions (red arrows) observed for EntA_Opt. Monoisotopic ions observed for EntA_Opt corresponding to the species (**b**) [M-4H+1O+4H(4)C(4)O(3)+6H]^+6^ (*m/z* 874.5739), (**c**) [M-4H+1O+4H(4)C(4)O(3)+5H]^+5^ (*m/z* 1049.2927), (**d**) [M-1H+1O+4H(4)C(4)O(3)+4H]^+4^ (*m/z* 1312.1118), (**e**) [M+1O+2H(4)C(4)O(3)+5H]^+5^ (*m/z* 1010.0739).

**
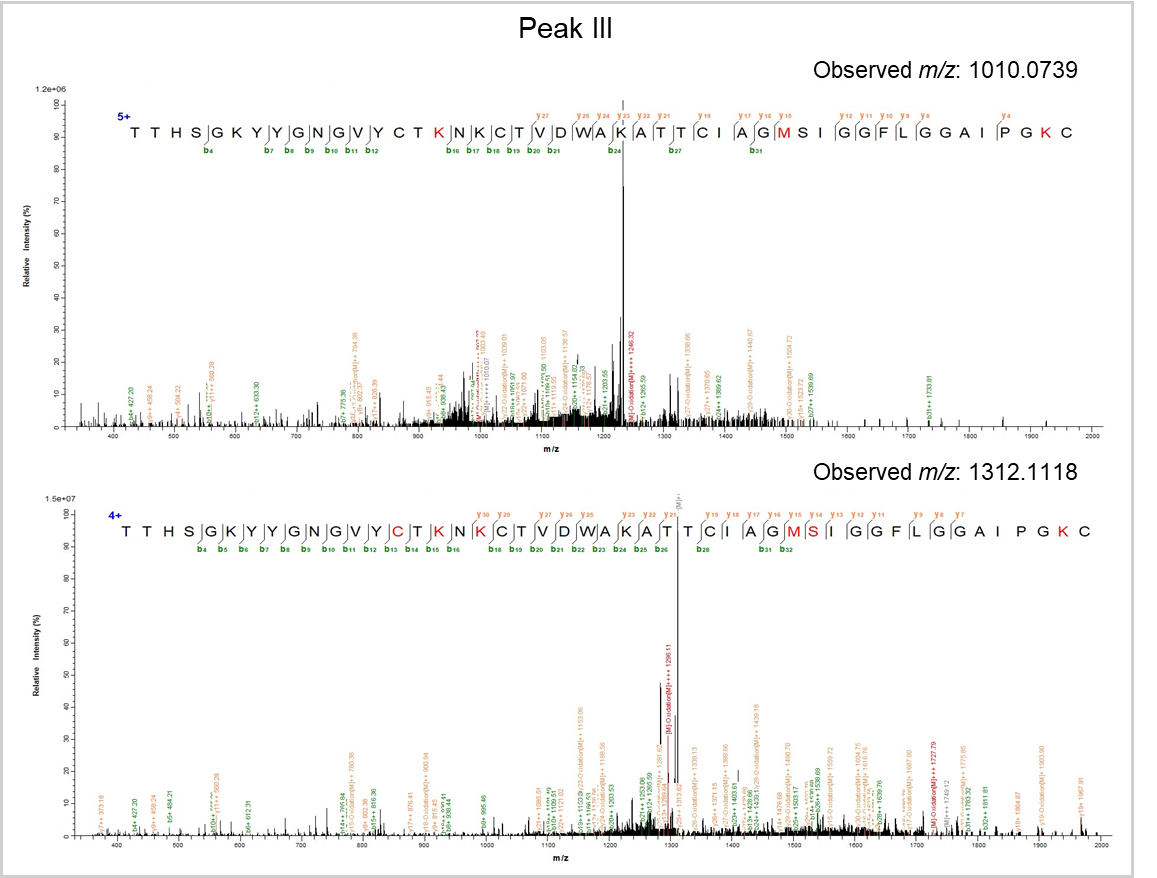
**

**Fig. S25** Tandem mass spectrometry of the monoisotopic parent ion from the peptide envelopes observed at peak III (retention time 21.19 min). Collision-induced peptide fragmentation spectra confirmed the EntA_Opt peptide sequence at peak III without disulphide bonds.


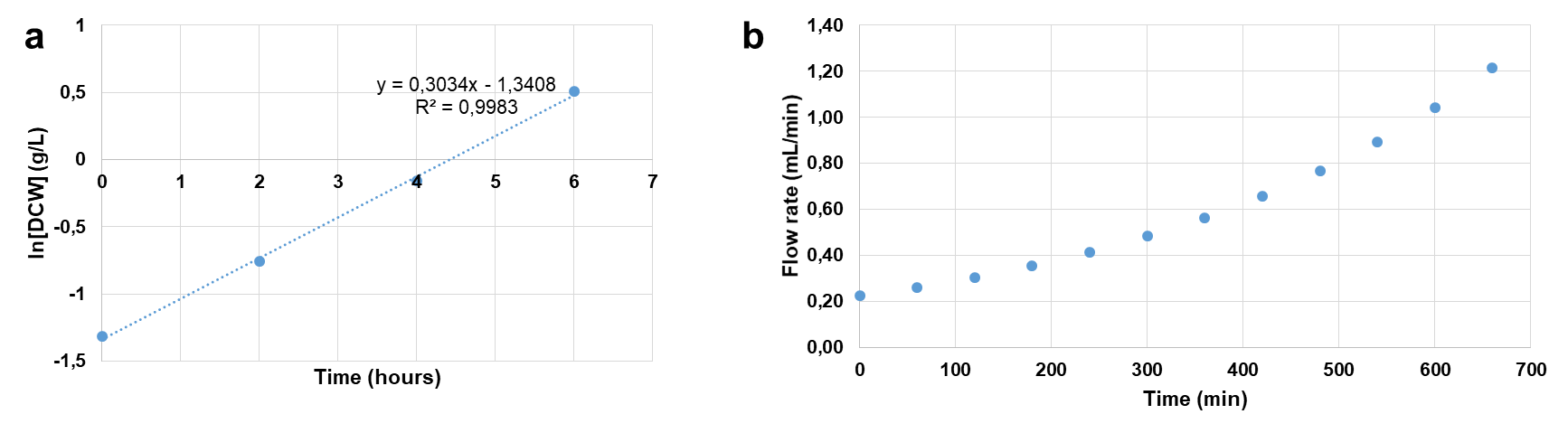


**Fig. S26** Calculations of the maximal specific growth rate (**a**) of Y294[MFα1-EntA_Opt] during the exponential growth phase of the batch cultivation and (**b**) the substrate feed profile for the fed-batch cultivation.

**Table S2:** Parameters for calculating the substrate feed rate for the fed-batch cultivation

|  | Value | Unit |
| --- | --- | --- |
| *µ_max_* | 0.30 | 1/h |
| % of *µ_max_* | 50% |  |
| *µ* | 0.154 | 1/h |
| *µ* | 0.003 | 1/min |
| *m_s_* | 8.33E-05 | g/g.min |
| *S_0_* | 100 | g/L |
| *Y_x/s_* | 0.07 | g/g |
| Step | 1 | min |
| Cell conc batch | 1.52 | g/L |
| Batch volume | 0,4 | L |
| *X_0_V_0_* | 0.608 | g |
| *t_0_* | 0 | h |
| Starting Volume | 0.4 | L |

**Table S3:** Glucose feed profile during fed-batch cultivation

| Time (min) | Time  (hours) | F (mL/min) | Volume fed/hour |
| --- | --- | --- | --- |
| 0 | 0.0 | 0.22 |  |
| 60 | 1 | 0.26 | 13.44 |
| 120 | 2 | 0.30 | 15.64 |
| 180 | 3 | 0.35 | 18.25 |
| 240 | 4 | 0.41 | 21.29 |
| 300 | 5 | 0.48 | 24.83 |
| 360 | 6 | 0.56 | 28.97 |
| 420 | 7 | 0.66 | 33.79 |
| 480 | 8 | 0.77 | 39.42 |
| 540 | 9 | 0.89 | 45.98 |
| 600 | 10 | 1.04 | 53.64 |
| 660 | 11 | 1.22 | 62.57 |
|  |  |  | 72.99 |


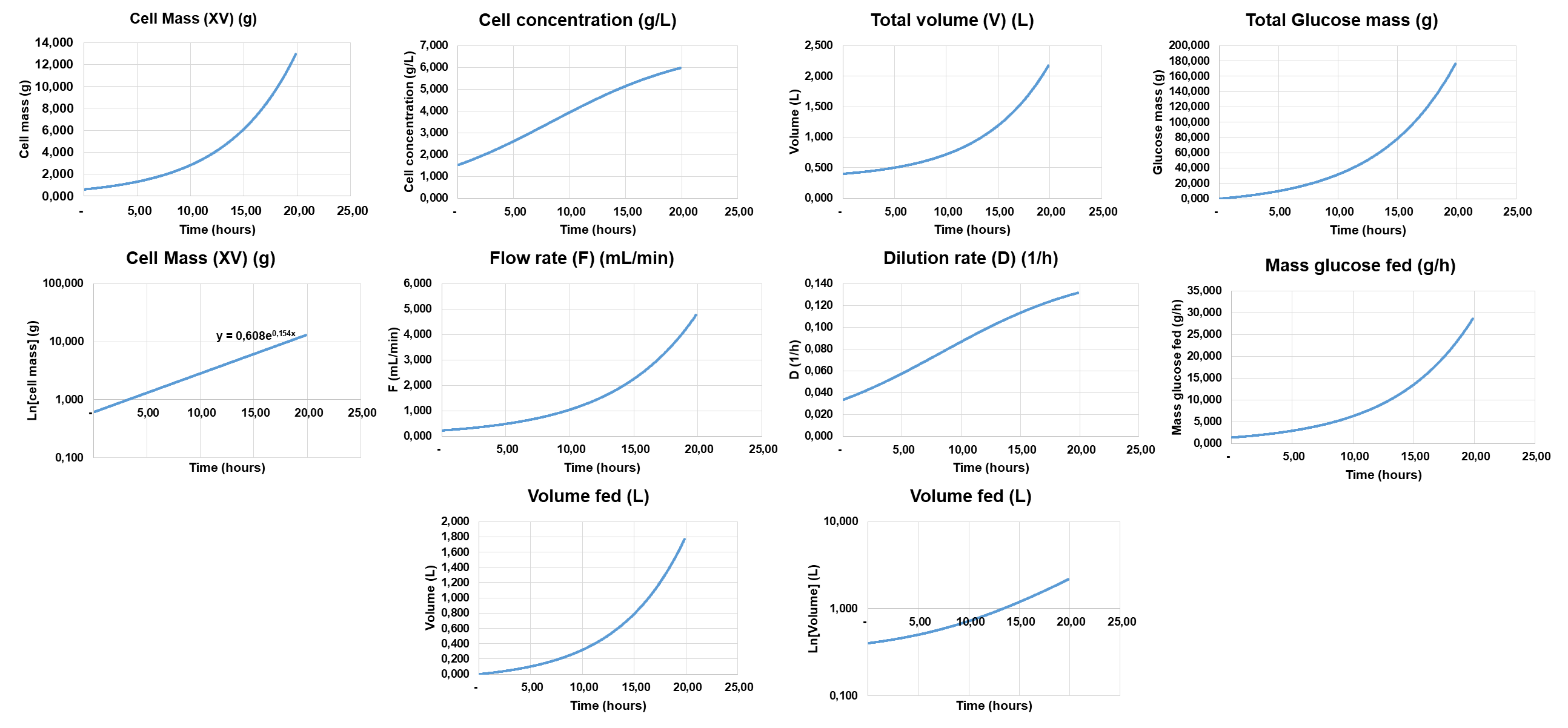


**Fig. S27** All parameters related to the fed-batch model for exponential feed.


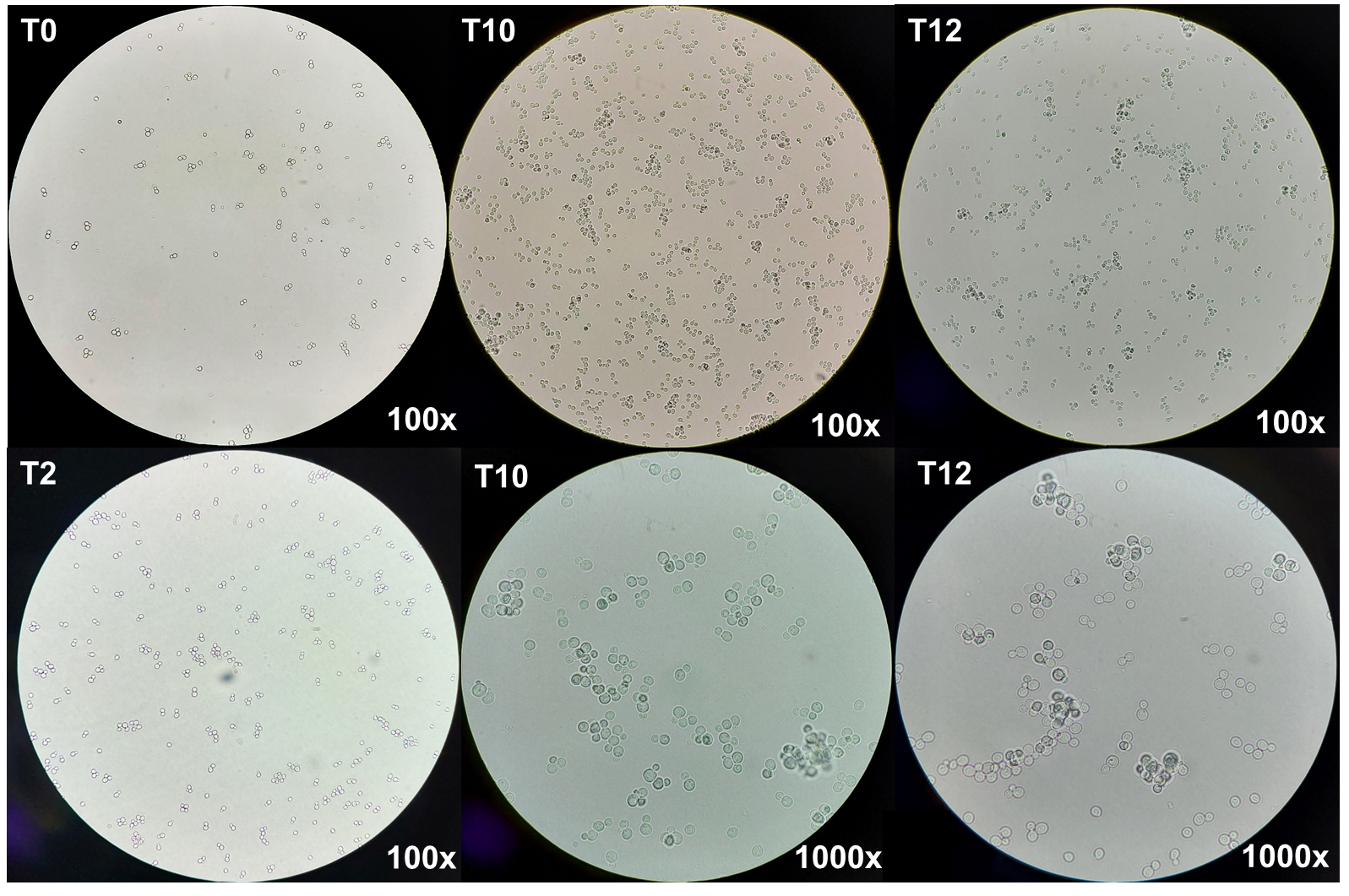


**Fig. S28** Light microscope images of the yeast cells from the fed-batch cultivation at times 0 hours, 2 hours, 10 hours and 12 hours at different magnifications. At time T10 and T12 the yeast cells formed clumps.


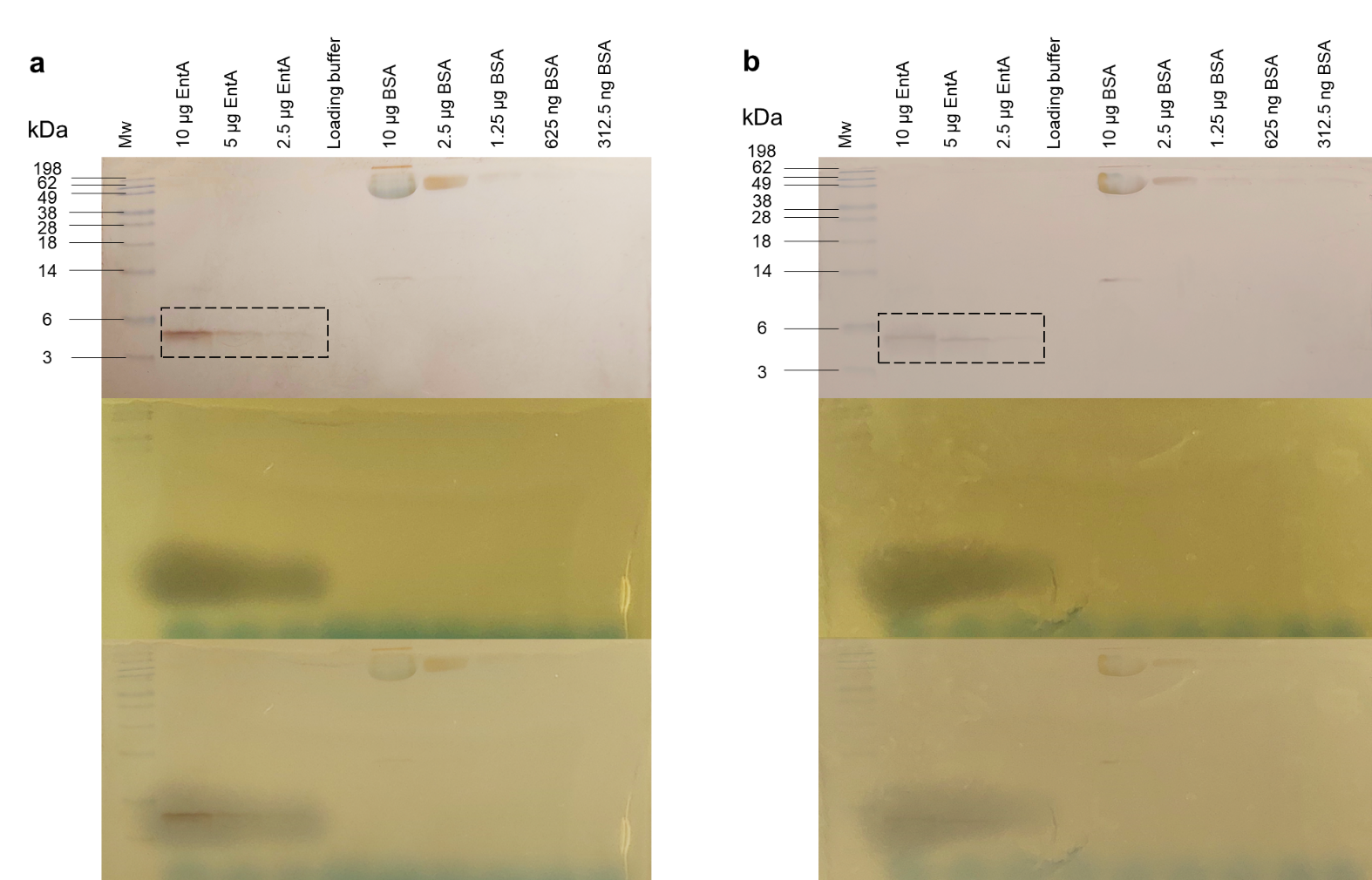


**Fig. S29** Tricine SDS-Page analysis and overlay of Sep-Pak C18-purified enterocin A (EntA_Opt) produced during batch (**a**) and fed-batch (**b**) cultivation in bioreactors. To confirm purity, 10 µg – 2.5 µg of the purified EntA was loaded onto the gel and bands corresponding to the size of enterocin A were detected after silver staining. Antilisterial activity was detected for EntA. A serial dilution of bovine serum albumin (BSA) of the same concentrations as the peptide was loaded onto the gel as a control. No larger bands were detected from the purified peptide samples, indicating at least 95% purity.
